# Modulation of *sol* mRNA expression by the long non-coding RNA Assolrna in *Clostridium saccharoperbutylacetonicum* affects solvent formation

**DOI:** 10.3389/fgene.2022.966643

**Published:** 2022-08-11

**Authors:** Saskia Tabea Baur, Anja Poehlein, Niklas Jan Renz, Stefanie Karolina Hollitzer, José David Montoya Solano, Bettina Schiel-Bengelsdorf, Rolf Daniel, Peter Dürre

**Affiliations:** ^1^ Institute of Microbiology and Biotechnology, University of Ulm, Ulm, Germany; ^2^ Department of Genomic and Applied Microbiology and Göttingen Genomics Laboratory, Institute of Microbiology and Genetics, Georg-August University of Göttingen, Göttingen, Germany

**Keywords:** antisense RNA, *Clostridium saccharoperbutylacetonicum*, stabilizing antisense RNA, Assolrna, ABE fermentation, *sol* operon

## Abstract

Solvents such as butanol are important platform chemicals and are often produced from petrochemical sources. Production of butanol and other compounds from renewable and sustainable resources can be achieved by solventogenic bacteria, such as the hyper-butanol producer *Clostridium saccharoperbutylacetonicum*. Its *sol* operon consists of the genes encoding butyraldehyde dehydrogenase, CoA transferase, and acetoacetate decarboxylase (*bld*, *ctfA*, *ctfB*, *adc*) and the gene products are involved in butanol and acetone formation. It is important to understand its regulation to further optimize the solvent production. In this study, a new long non-coding antisense transcript complementary to the complete *sol* operon, now called Assolrna, was identified by transcriptomic analysis and the regulatory mechanism of Assolrna was investigated. For this purpose, the promoter-exchange strain *C. saccharoperbutylacetonicum* ΔP_*asr*_ *::*P_asr_^**^ was constructed. Additionally, Assolrna was expressed plasmid-based under control of the native P_*asr*_ promoter and the lactose-inducible P_*bgaL*_ promoter in both the wild type and the promoter-exchange strain. Solvent formation was strongly decreased for all strains based on *C. saccharoperbutylacetonicum* ΔP_*asr*_ *::*P_asr_^**^ and growth could not be restored by plasmid-based complementation of the exchanged promoter. Interestingly, very little *sol* mRNA expression was detected in the strain *C. saccharoperbutylacetonicum* ΔP_*asr*_ *::*P_asr_^**^ lacking Assolrna expression. Butanol titers were further increased for the overexpression strain *C. saccharoperbutylacetonicum* [pMTL83151_*asr*_P_*bgaL*_] compared to the wild type. These results suggest that Assolrna has a positive effect on *sol* operon expression. Therefore, a possible stabilization mechanism of the *sol* mRNA by Assolrna under physiological concentrations is proposed.

## Introduction

*Clostridium saccharoperbutylacetonicum* was originally isolated as hyper-butanol producer and was used for industrial butanol and acetone production *via* fermentation in Japan (Hongo, 1959; Ogata and Hongo, 1979). Nowadays, butanol is derived from petroleum (Kotsanopoulos et al., 2019), but it can also be produced *via* fermentation of different substrates (Birgen et al., 2019). In recent years, research on fuel production by fermentation of solventogenic bacteria comes back into focus. The solvent formation (*sol*) operon of *C. saccharoperbutylacetonicum* is encoding butyraldehyde dehydrogenase (*bld*), CoA transferase (*ctfA* and *ctfB*), and acetoacetate decarboxylase (*adc*) is located on the chromosome (Cspa_c56880–Cspa_c56910) (Poehlein et al., 2014; Poehlein et al., 2017). The enzymes encoded there are involved in butanol and acetone formation (Fischer et al., 1993; Kosaka et al., 2007; Poehlein et al., 2014). Solvent-producing clostridia show a bi-phasic growth consisting of an acidogenic and a solventogenic growth phase (Bahl et al., 1982). The switch from acidogenesis to solventogenesis is tightly controlled (Kosaka et al., 2007; Dürre, 2014; Atmadjaja et al., 2019; Yang et al., 2020). Sequencing revealed that *C. saccharoperbutylacetonicum* contains a chromosome of 6.53 Mbp and a megaplasmid harboring 136 kbp (Poehlein et al., 2014). The strain can be cured from the megaplasmid resulting in a slight increase in solvent formation and an enhanced transformation efficiency (Gu et al., 2019), but efficient transformation can also be achieved when it is still containing the megaplasmid (Atmadjaja et al., 2019; Baur et al., 2022). Furthermore, the strain can sporulate which can be uncoupled from solvent formation by a single nucleotide polymorphism (Atmadjaja et al., 2019).

Acid production was recently optimized in *C. saccharoperbutylacetonicum* (Baur et al., 2022), since it can be used for large-scale production of high-value compounds such as butyl esters from sustainable resources (Feng et al., 2021). In addition to that, *C*. *saccharoperbutylacetonicum* can be used as production strain for other recombinant products like hexanol or 1,3-butanediol (Grosse-Honebrink et al., 2021; Wirth and Dürre, 2021).

Transcriptomic data of *C. saccharoperbutylacetonicum* revealed a high coverage of the genome with *cis-*antisense RNAs (asRNAs), for example complementary to the complete *sol* operon (Baur, 2022). Native asRNAs can affect transcription, translation, and RNA degradation positively and negatively using different mechanisms (Brantl, 2007; Romby and Charpentier, 2010; Georg and Hess, 2011; Stazic et al., 2011; Morris and Mattick, 2014; Cho and Kim, 2015; Papenfort and Vanderpool, 2015). They can be present overlapping either the 5′ end (divergent), the 3′ end (convergent), internally, or covering the whole gene or operon (Georg and Hess, 2011). Interaction of asRNA and mRNA can lead to negative regulation of the mRNA by prevention of formation of an antiterminator structure (André et al., 2008), inhibition of binding of ribosomes to ribosomal binding sites (RBS) or start codons (Huntzinger et al., 2005; Brantl, 2007), or recruiting of RNases such as RNase E, J1, or J2 for degradation of the RNA-RNA duplex (Condon and Bechhofer, 2011; Georg and Hess, 2011; Stazic et al., 2011). Besides that, it can also lead to positive regulation *via* increase of RNA stability by altering the secondary structure in a way that occluded RBSs and start codons are freely available to ribosomes, or directed cleavage of the mRNA in a way that the secondary structure of the resulting fragments is more stable than the whole mRNA (Brantl, 2007; Opdyke et al., 2011; Papenfort and Vanderpool, 2015). Furthermore, binding of the asRNA can lead to masking of recognition sites of RNases E, J1, J2, and others, thereby preventing degradation of the mRNA (Thomason and Storz, 2010; Cho and Kim, 2015).

In this study, the long non-coding RNA Assolrna was discovered during analysis of transcriptomic data. Furthermore, its regulatory function was characterized and a model of the regulation of the *sol* operon of *C*. *saccharoperbutylacetonicum* by Assolrna is suggested. The understanding of its regulatory role is important for further improvements of acid and solvent formation for industrial use since several other asRNAs with similar expression patterns were identified in the transcriptomic data of *C*. *saccharoperbutylacetonicum*.

## Materials and methods

### Bacterial strains and cultivation conditions

*E. coli* strains were cultured at 37°C in LB medium (Green and Sambrook, 2012) or SOB medium (Hanahan, 1983) with appropriate antibiotics (30 µg ml^−1^ chloramphenicol, 250 µg ml^−1^ erythromycin, or 10 µg ml^−1^ tetracycline). *C*. *saccharoperbutylacetonicum* strains were cultured at 30°C in clostridial growth medium (CGM) for transformation or pre-cultures as previously described (Wirth and Dürre, 2021), and optimized, synthetic medium (OMS) was used for all growth experiments as described (Wirth and Dürre, 2021). If appropriate, antibiotics were added (75 µg ml^−1^ thiamphenicol or 10 µg ml^−1^ clarithromycin). All strains and plasmids used are listed in Supplementary Table S1.

### Isolation of RNA

Isolation of RNA from 2-ml and 50-ml samples was carried out using TRI reagent and chloroform/isoamyl alcohol (24:1). DNA digestion of RNA from 50-ml samples was performed using DNase I (Invitrogen™, a Thermo Fisher Scientific Inc. brand, Waltham, MA, United States). Afterwards, RNA was purified using phenol/chloroform/isoamyl alcohol (25:24:1) precipitation as described by Montoya Solano (Montoya Solano, 2013). In contrast, DNA digestion of RNA isolated from 2-ml samples was carried out using the Ambion^®^ TURBO DNA-free™ Kit (Invitrogen™, a Thermo Fisher Scientific Inc. brand, Waltham, MA, United States) according to manufacturer’s instructions. Remains of DNA were checked by PCR using the primers for the subsequent (q)RT-PCR.

### Transcriptomic data and analysis using TraV

The 50-ml samples for first transcriptome analysis were taken from one 200-ml OMS culture for the acidogenic growth phase (after 23 h, OD_600_ of 0.40) and from another 200-ml OMS culture for the solventogenic growth phase (after 40 h, OD_600_ of 5.65). Three technical replicates were performed by preparing RNA from three 50-ml samples from the same culture. Remaining genomic DNA was removed by digesting with TURBO DNase (Invitrogen™, a Thermo Fisher Scientific Inc., brand, Waltham, MA, United States). The Ribo-Zero magnetic kit (Epicentre Biotechnologies, Madison, WI, United States) was used to reduce the amount of rRNA-derived sequences. For sequencing, the strand-specific cDNA libraries were constructed with a NEBNext Ultra directional RNA library preparation kit for Illumina (New England Biolabs GmbH, Frankfurt am Main, Germany). To assess quality and size of the libraries, samples were run on an Agilent Bioanalyzer 2100 using an Agilent High Sensitivity DNA Kit as recommended by the manufacturer (Agilent Technologies Inc., Waldbronn, Germany). Concentration of the libraries were determined using the Qubit^®^ dsDNA HS Assay Kit as recommended by the manufacturer (Life Technologies GmbH, Darmstadt, Germany). Sequencing was performed by using the Genome Analyzer Iix maschine (Illumina Inc., San Diego, CA, United States) for sequencing in the paired-end mode and running 2 × 75 cycles. For quality filtering and removing of remaining adaptor sequences, Trimmomatic-0.39 (Bolger et al., 2014) and a cutoff phred-33 score of 15 were used. The mapping of the remaining sequences was performed with the Bowtie (version 2) program (Langmead and Salzberg, 2012) using the implemented end-to-end mode, which requires that the entire read align from one end to the other. First, surviving reads were mapped against a database consisting of tRNA and rRNA sequences of *C. saccharoperbutylacetonicum* N1-4(HMT) (Poehlein et al., 2014) and unaligned reads were subsequently mapped against the genome of *C. saccharoperbutylacetonicum* N1-4(HMT). Differential expression analyses were performed with the BaySeq program (Mortazavi et al., 2008). Genes with log_2_(fold change) in expression of ≥ 2.0 or ≤−2.0, a likelihood value of ≥ 0.9, and an adjusted *p* value of ≤ 0.05 were considered differentially expressed. The *p* value was corrected by the false discovery rate (FDR) based on the Benjamini-Hochberg procedure. The raw reads have been deposited in the National Center for Biotechnology Information’s (NCBI) Sequence Read Archive (SRA) under accession no. SRP357609.

For further investigation of Assolrna, a new transcriptome was prepared from biological triplicates. 1- and 2-ml samples were drawn from 50-ml OMS cultures during a growth experiment. Cell pellets from biological triplicates from the early exponential growth phase (after 12 h, OD_600_ of approximately 0.70), from after the butyrate peak (after 24 h, OD_600_ of approximately 3.6), from after the acetate peak (after 29.5 h, OD_600_ of approximately 6.1), and from the stationary growth phase (after 52 h, OD_600_ of approximately 6.7). Harvested cells were suspended in 800 µl RLT buffer (RNeasy Mini Kit, Qiagen N.V., Hilden, Germany) with β-mercaptoethanol (10 µl ml^−1^) and cell lysis was performed using a laboratory ball mill. Subsequently, 400 µl RLT buffer (RNeasy Mini Kit, Qiagen) with β-mercaptoethanol (10 µl ml^−1^) and 1,200 µl 96 % (v/v) ethanol were added. For RNA isolation, the RNeasy Mini Kit (Qiagen N.V., Hilden, Germany) was used as recommended by the manufacturer, but instead of RW1 buffer RWT buffer (Qiagen N.V., Hilden, Germany) was used to isolate RNAs smaller 200 nt. To determine the RNA integrity number (RIN), the isolated RNA was run on an Agilent Bioanalyzer 2100 using an Agilent RNA 6000 Nano Kit as recommended by the manufacturer (Agilent Technologies Inc., Waldbronn, Germany). Library preparation and sequencing was performed as described above, but with the following modifications: The Illumina Ribo-Zero plus rRNA Depletion Kit (Illumina Inc., San Diego, CA, United States) was used to reduce the amount of rRNA-derived sequences. For preparation of strand-specific cDNA libraries, the NEBNext Ultra II directional RNA library preparation kit for Illumina and the NEBNext Multiplex Oligos for Illumina (96) (New England Biolabs GmbH, Frankfurt a. M., Germany) were used. Sequencing was performed on the NovaSeq 6000 instrument (Illumina Inc., San Diego, CA, United States) using NovaSeq 6000 SP Reagent Kit v 1.5 (100 cycles) and the NovaSeq XP 2-Lane Kit v 1.5 for sequencing in the paired-end mode and running 2 × 50 cycles. The mapping against the reference genomes was performed with Salmon (v 1.5.2) (Patro et al., 2017). As mapping backbone, a file that contains all annotated transcripts excluding rRNA genes and the whole genome of the references as decoy was prepared with a k-mer size of 11. Decoy-aware mapping was done in selective-alignment mode with “–mimicBT2,” “–disableChainingHeuristic,” and “–recoverOrphans” flags as well as sequence and position bias correction. For –fldMean and –fldSD, a value of 325 and 25 was used, respectively. The quant.sf files produced by Salmon were subsequently loaded into R (v 4.0.3) (R Core Team, 2020) using the tximport package (v 1.18.0) (Soneson et al., 2015). DeSeq2 (v 1.30.0) (Love et al., 2014) was used for normalization of the reads and foldchange-shrinkages were also calculated with DeSeq2 and the apeglm package (v 1.12.0) (Zhu et al., 2019). Genes with a log_2_(fold change) of expression ≥ 2.0 or ≤ −2.0 and an adjusted *p* value of ≤ 0.05 were considered differentially expressed. The asRNA complementary to the *sol* operon, now called Assolrna, as well as the potential TSS corresponding to P_*asr*-is_ was identified using TraV (Dietrich et al., 2014). *In silico* predictions regarding P_*asr*-is_ were carried out using SAPPHIRE (Coppens and Lavigne, 2020) and Neural Network Promoter Prediction (Reese, 2001). Furthermore, TraV was used to calculate nucleotide activity per kilobase of exon model per million mapped reads (NPKM) from transcriptional activity (ta) (Dietrich et al., 2014). Raw reads have been deposited in the NCBI’s SRA under accession no. SRP343145.

### Primer extension

The RNA for the primer extension (PEX) was prepared from 50-ml samples from a *C. saccharoperbutylacetonicum* N1-4(HMT) culture. PEX were performed according to Montoya Solano (2013). All primers used are listed in Supplementary Table S2.

### Construction of plasmids

All primers used for amplification of inserts are listed in Supplementary Table S2. For the PEX experiments, the region probably containing the TSS (of P_*asr*_) was amplified using ReproFast proofreading polymerase (Genaxxon bioscience GmbH, Ulm, Germany) and primers Fwd_1_solregpotRNA and Rev_1_solregpotRNA, Fwd_1_solregpotRNA and Rev_2_ solregpotRNA, Fwd_1_solregpotRNA and Rev_3_solregpot RNA, Fwd_2_solregpotRNA and Rev_1_solregpotRNA, and Fwd_2_solregpotRNA and Rev_2_solregpotRNA. The yielded fragments were purified from gel using the NucleoSpin Gel and PCR Clean-up kit (Macherey-Nagel GmbH & Co. KG, Düren, Germany) and ligated into the vector pDrive using the Qiagen^®^ PCR Cloning Kit (Qiagen N.V., Hilden, Germany) according to manufacturers’ instructions. The ligation approach was transformed into *E. coli* XL1-Blue MRF’. Colony PCR was carried out with primers M13F and M13R. Clones showing the expected fragment length were inoculated, plasmid was prepared using the Zyppy Plasmid Miniprep Kit (ZYMO Research, Freiburg, Germany), and sent for sequencing.

The vector for allelic exchange was constructed by plasmid preparation from *E. coli* CA434 [pMTL83251] and *E. coli* DH5α [pMTL-SC7515] using the Zyppy Plasmid Miniprep Kit (ZYMO Research, Freiburg, Germany) according to the manufacturer’s instructions. The plasmids were linearized using *Fse*I with CutSmart Buffer by New England Biolabs GmbH (Frankfurt am Main, Germany) and purified using the NucleoSpin Gel and PCR Clean-up kit (Macherey-Nagel GmbH & Co. KG, Düren, Germany) according to manufacturers’ instructions. Afterwards, the linearized plasmids were digested with *Pme*I and Buffer B (New England Biolabs GmbH, Frankfurt am Main, Germany) with Shrimp Alkaline Phosphatase (Applied Biosystems™, a Thermo Fisher Scientific Inc., brand, Waltham, MA, United States) added to the reaction containing pMTL-SC7515 according to manufacturers’ instructions. Both reactions were applied on an agarose gel and the expected fragments (*ermB* from pMTL83251, backbone from pMTL-SC7515) were purified from gel using the NucleoSpin Gel and PCR Clean-up kit (Macherey-Nagel GmbH & Co., KG, Düren, Germany) according to manufacturer’s instructions. The fragments were ligated using T4 ligase (Thermo Fisher Scientific Inc., Waltham, MA, United States) and transformed into *E. coli* DH5α. Colony PCR was performed using the primers ermC-FseI_fwd and ermC-nachPmeI_rev. Positive clones were inoculated, and the plasmid prepared using the Zyppy Plasmid Miniprep Kit (ZYMO Research, Freiburg, Germany), and verified using control digestion. The successfully constructed plasmid was designated pMTLSC7515-Em.

The cassette for the knock-out of P_*asr*_ through exchange by P_asr_** was constructed by amplifications of the fragments LHA and RHA using the primers LHA_fwd_PromRNA, LHA_rev_ PromRNA, RHA_fwd_PromRNA, and RHA_rev_PromRNA for the PCR with ReproFast space proofreading polymerase (Genaxxon bioscience GmbH, Ulm, Germany). Afterwards, a splicing overlap extension PCR was performed using the fragments LHA and DHA as template with primers LHA_fwd_PromRNA and RHA_rev_PromRNA. The resulting fragment was purified from the gel using the Zymoclean Gel DNA Recovery kit (ZYMO Research, Freiburg, Germany) and ligated into the vector pDrive using the Qiagen^®^ PCR Cloning Kit (Qiagen N.V., Hilden, Germany) according to manufacturers’ instructions resulting in plasmid pDrive_recA. The correct clone was identified by colony PCR using primers M13F and M13R and sent for sequencing. The plasmids pDrive_recA and pMTLSC7515-Em were digested using *PmeI* and Buffer B (New England Biolabs GmbH, Frankfurt am Main, Germany). Shrimp Alkaline Phosphatase (Applied Biosystems™, a Thermo Fisher Scientific Inc. brand, Waltham, MA, United States) was added to digestion of pMTLSC7515-Em. The desired fragments (knock-out cassette from pDrive_recA, backbone from pMTLSC7515-Em) were purified from gels using the Zymoclean Gel DNA Recovery kit (ZYMO Research, Freiburg, Germany) according to manufacturer’s instructions. Ligation of the fragments was performed using the T4 ligase (Thermo Fisher Scientific Inc., Waltham, MA, United States) and transformed into *E. coli* XL-1 Blue MRF’. Colony PCR with primers LHA_fwd_PromRNA and RHA_rev_PromRNA was carried out to identify positive clones. The resulting plasmid pMTLSC7515-Em-recA was sent for sequencing and later renamed to pMTL-PromoterRNA.

Ligation of complementation and overexpression plasmid was carried out using In-Fusion^®^ HD Cloning Plus (Takara Bio Inc., Kusatsu, Shiga, Japan) according to manufacturer’s instructions. Digestions were performed using Fast Digest enzymes (Thermo Fisher Scientific Inc., Waltham, MA, United States) according to manufacturer’s instructions.

The plasmids pMTL_Komp_P_*asr*_, pMTL_Komp_P_*asr*_T , and pMTL83151_*asr*_P_*bgaL*_ were constructed as described by Baur (2022).

The plasmid pMTL83151_Komp_P_*asr*_T was digested using *Xho*I and *Sal*I to yield the backbone of pMTL83151_asADC_P_*asr*_T. The insert was amplified from genomic DNA of *C*. *saccharoperbutylacetonicum* using the primers asADC_Pasr_fwd and asADC_Pasr_rev. Ligation was performed using In-Fusion^®^ HD Cloning Plus (Takara Bio Inc., Kusatsu, Shiga, Japan) according to manufacturer’s instructions. An overview on all constructed plasmids is given in Supplementary Table S1.

### Construction of recombinant *E. coli* and *C. saccharoperbutylacetonicum* strains

Chemically competent *E*. *coli* cells were prepared and transformed as previously described by Inoue et al. (1990). *C. saccharoperbutylacetonicum* was electro-transformed as described by Atmadjaja et al. (2019) with slight modifications as described by Wirth and Dürre (2021).

Exchange of the promoter region P_*asr*_ with P_asr_^**^ in *C. saccharoperbutylacetonicum* was performed using the allelic exchange system as described by Ehsaan et al. (2016). In brief, *C. saccharoperbutylacetonicum* was transformed with pMTL-PromoterRNA harboring *codA* as a counter selection marker when 5-fluorocytosine is used. Genomic DNA of all clones was prepared using the MasterPure™ Gram-Positive DNA Purification Kit (Lucigen Corp. Middleton, WI, United States) according to manufacturer’s instructions. All clones were tested for genomic integration using the primers pMTL-PR_fwd and assolrna-genom_rev as well as assolrna-genom_fwd and pMTL-PR_rev. When genomic integration was verified, CGM was inoculated for plating on CGM agar containing 5-fluorocytosine (100 µg/ml^−1^). Colonies were picked, genomic DNA was isolated, and successful excision of the plasmid was tested using primers pMTL-PR_fwd and pMTL-PR_rev in a PCR. When excision was detected, the P_*asr*_/P_asr_^**^ region was amplified using the primers assolrna-genom_fwd and assolrna-genom_rev, fragments were purified using the DNA Clean & Concentrator Kit (ZYMO Research, Freiburg, Germany) and sent for sequencing. The successfully constructed promoter exchange strain was designated *C. saccharoperbutylacetonicum* ΔP_*asr*_ *::*P_asr_^**^.

### Verification of strains

The polymerases Platinum™ II Hot-Start Green PCR Master Mix, Platinum™ SuperFi™ PCR Master Mix, Platinum™ SuperFi™ II PCR Master Mix (Invitrogen™, a Thermo Fisher Scientific Inc., brand, Waltham, MA, United States), Phusion Green High-Fidelity DNA Polymerase (Thermo Scientific™, a Thermo Fisher Scientific Inc., brand, Waltham, MA, United States), CloneAmp™ HiFi PCR Premix (Takara Bio Inc., Kusatsu, Shiga, Japan), and ReproFast proofreading Polymerase (Genaxxon bioscience GmbH, Ulm, Germany) were used for amplification of fragments according to manufacturers’ instructions. Sequencing reactions were performed by GATC Biotech AG (now part of Eurofins Scientific SE, Luxembourg, Luxembourg) or GENEWIZ (by Azenta Life Sciences, South Plainfield, NJ, United States).

For verification of plasmids, the plasmids were digested using appropriate restriction enzymes or tested *via* colony PCR and subsequently sent for sequencing. Verification of *C. saccharoperbutylacetonicum* strains was carried out by amplification and sequencing of 16S rDNA, P_*asr*_/P_asr_^**^ promoter region, and transformation of genomic DNA into chemically competent *E. coli* with subsequent picking of colonies, purification of plasmids (Zyppy™ Plasmid Miniprep Kit, ZYMO Research, Freiburg, Germany), and restriction digestion of purified plasmids (Thermo Fisher Scientific Inc., Waltham, MA, United States) according to manufacturer’s instructions.

### Determination of product formation and glucose consumption

Glucose consumption, lactate formation, and presence of lactose was quantified using high-pressure liquid chromatography as described by Wirth and Dürre (2021). Formation of the products acetate, butyrate, acetone, ethanol, and butanol was measured using gas chromatography as described before (Wirth and Dürre, 2021).

### Statistical analysis using R

Statistical analysis of the product formation of interest, i.e., butanol and acetone, was carried out using R v 4.0.3 (R Core Team, 2020). First, an ANOVA was calculated followed by Tukey multiple comparison of means for all tested strain and concentrations at the end of the growth experiment shown in Table 2.

### RT-PCR and qRT-PCR

Reverse transcription for RT-PCR was performed with DNA-free RNA of *C. saccharoperbutylacetonicum* aReverse transcription PCRnd SuperScript™ III reverse transcriptase (Invitrogen™, a Thermo Fisher Scientific Inc. brand, Waltham, MA, United States) according to manufacturer’s instructions with primer RT_asr_fwd. The PCR was performed using 2 μl of the solution with the newly synthesized cDNA, the primers RT_asr_fwd and RT_ig670_rev), and Phusion Green High-Fidelity DNA Polymerase (Thermo Scientific™, a Thermo Fisher Scientific Inc., brand, Waltham, MA, United States)Reverse transcription PCR.

Strand specific reverse transcription for qRT-PCR was performed using DNA-free RNA and Maxima H Minus Reverse Transcriptase (Thermo Scientific™, a Thermo Fisher Scientific Inc., brand, Waltham, MA, United States) according to manufacturer’s instructions using a 20 μM primer mix consisting of 16SR_qPCR (Wang et al., 2008) with either AdhEF_qPCR or AdcR_qPCR. Primer efficiency tests and quantitative PCRs were performed using matching primers and 1:2 serial dilutions of *C. saccharoperbutylacetonicum* genomic DNA (starting from 100 ng) with PowerUp™ SYBR™ Green Master Mix (Applied Biosystems™, a Thermo Fisher Scientific Inc., brand, Waltham, MA, United States). The primer pairs 16SF_qPCR/16SR_qPCR, AdhEF_qPCR/AdhER_qPCR, and AdcF_qPCR/AdcR_qPCR were used for specific detection of 16S rRNA, *sol* mRNA, and Assolrna, respectively. Primer efficiency tests as well as quantitative PCR were performed by a CFX96 Touch™ Real-Time PCR Detection System (Bio-Rad Laboratories, Inc., Hercules, CA, United States) in a PCR 96-Well TW-MT-Plate sealed with Adhesive Clear qPCR Seals, Sheets (Biozym Scientific GmbH, Oldendorf, Germany). Primer efficiency was between 0.917 and 1.000, hence they are in the appropriate range (Schmittgen and Livak, 2008; Baur, 2022). 16S rRNA expression showed very little variability in contrast to *gyrB* expression and was, therefore, used as reference. Cycle threshold (C_*T*_) and runs were calculated using the CFX Manager™ Software Version 3.1 (Bio-Rad Laboratories, Inc., Hercules, CA, United States). C_*T*_ values were normalized to the ones of 16S rRNA and relative expression levels were calculated (Schmittgen and Livak, 2008).

## Results

### Discovery of Assolrna and identification of its promoter and terminator

**FIGURE 1:**
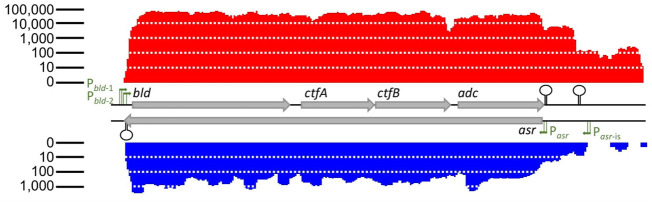
Expression levels of *sol* mRNA and Assolrna. Red, transcriptional activity of *sol* mRNA; blue, transcriptional activity of Assolrna; grey arrows, genes encoding butyraldehyde dehydrogenase (*bld*), CoA-transferase subunits α (*ctfA*) and β (*ctfB*), acetoacetate decarboxylase (*adc*) (Cspa_c56880 to Cspa_c56910), and Assolrna (*asr*). Transcriptional activity is the calculated base coverage from mapped reads (Dietrich et al., 2014). Green arrows with lines, promoters of respective genes depicting the −10 and −35 regions; stem-loop structures, terminators. There is a sharp decrease in transcriptional activity at the end of the displayed transcript, but no terminator was identified. Transcriptional activity at both ends of the figure were not cut off intentionally, they indeed ended there.

**FIGURE 2:**
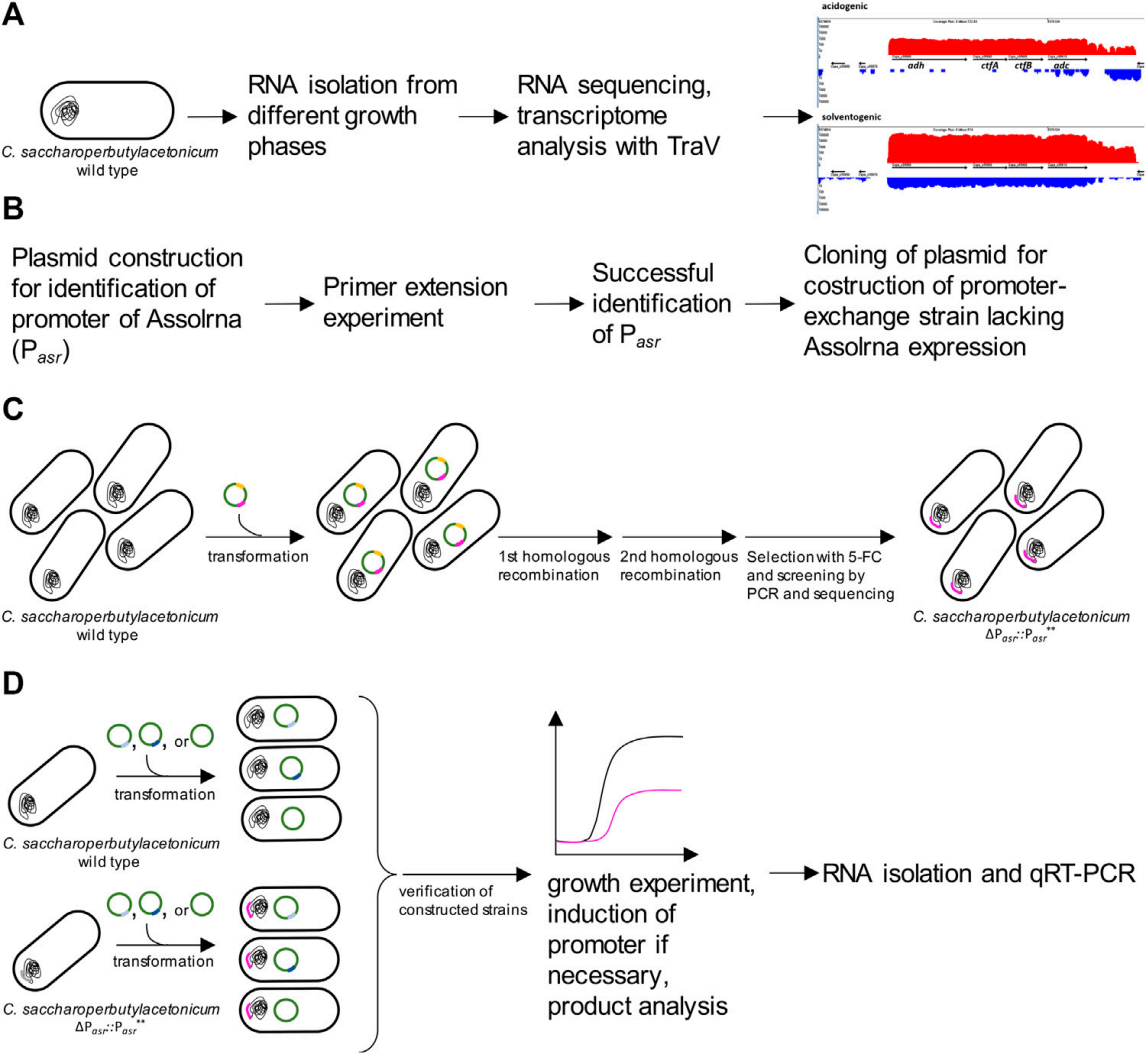
Overview of different experiments conducted in this study. **(A)** identification of Assolrna using TraV’s “antisense transcript search” algorithm (red showing transcripts of + strand, blue showing transcripts of—strand); **(B)** steps performed for identification of promoter of Assolrna; **(C)**, construction of *C. saccharoperbutylacetonicum* ΔP_*asr*_ *::*P_asr_^**^ using the plasmid pMTL-PromoterRNA harboring homologous regions from upstream and downstream of the identified P_*asr*_ promoter and a modified sequence for the promoter region (P_asr_^**^) (pink) as well as *codA* (yellow) as selection marker for 5-fluorocytosine (5-FC); **(D)** construction of plasmid based overexpression, complementation, and control strains (bright blue insert: pMTL83151_Komp_P_*asr*_T, dark blue insert: pMTL83151_*asr*_P_*bgaL*_, no insert: pMTL83151), growth experiment, and characterization of growth, product formation and transcription by qRT-PCR.

The *sol* operon of *C. saccharoperbutylacetonicum* was characterized by Kosaka and co-workers (Kosaka et al., 2007). They identified the promoters P_*bld*-1_ and P_*bld*-2_ of the *sol* operon consisting of the genes *bld*, *ctfA*, *ctfB*, and *adc* encoding butyraldehyde dehydrogenase, CoA transferase subunits α and β, and acetoacetate decarboxylase (Kosaka et al., 2007). Additionally, they provided evidence for transcription as operon (Kosaka et al., 2007). Strand-specific transcriptomic data performed as technical replicates revealed the presence of an asRNA complementary to the complete *sol* operon of *C. saccharoperbutylacetonicum* N1-4(HMT) (Figure 1). This was achieved using the software TraV and its included module “antisense transcript search” with manual examination of the data. The data showed transcription only during the solventogenic growth phase (Figure 2). Sequence analysis of the potential end of Assolrna and sequence comparison with SolB encoded in *Clostridium acetobutylicum* revealed an identical terminator sequence overlapping with P_*bld*-2_ in *C. saccharoperbutylacetonicum* as shown in Figure 1 (Jones et al., 2018; Baur, 2022). There were hints for a novel asRNA with possible stabilizing function, i.e., transcription of the asRNA at the same time as the *sol* mRNA. Therefore, further experiments were conducted to test the presence of the asRNA and investigate its possible regulatory role (Figure 2).

**FIGURE 3:**
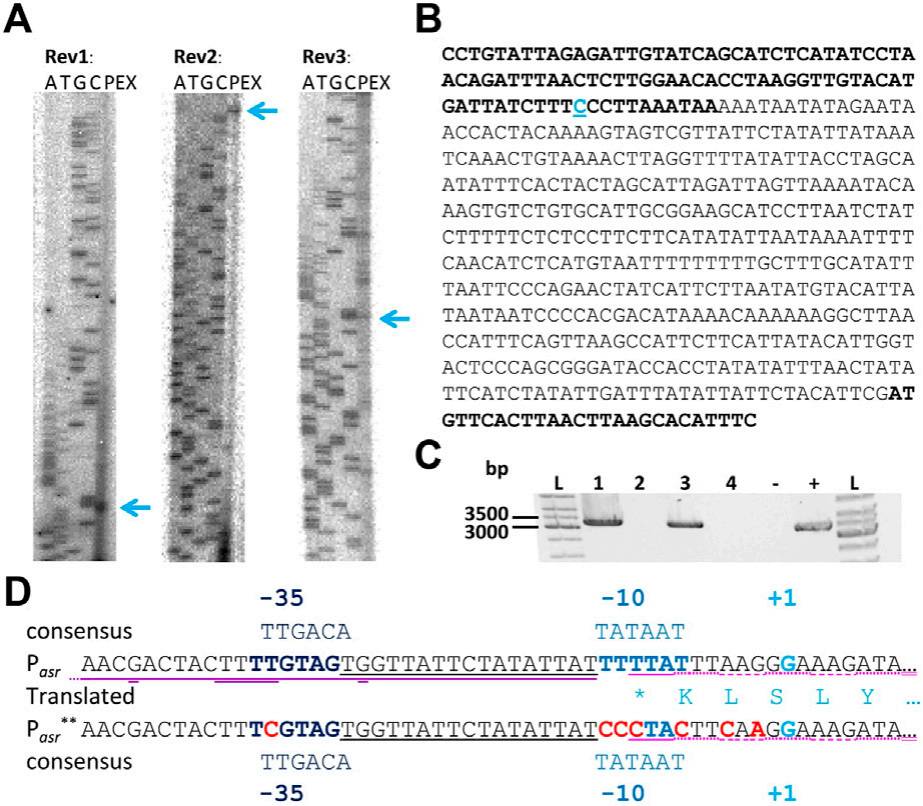
Primer extension experiments, TSS, promoter, and proof of length of Assolrna. **(A)** three independent primer extension experiments with associated sequencing reaction with primers Rev_1_solregpotRNA, Rev_2_solregpotRNA, and Rev_3_solregpotRNA (A, adenine; T, thymidine; G, guanine; C, cytosine; PEX, primer extension experiment) for determination of TSS of Assolrna; blue arrows, marked TSS; **(B)** sequence of fragment Fwd1/Rev2 containing the promoter area; blue C, marked TSS; bold font, end of coding region of Cspa_c56910 (*adc*) and start of coding region of Cspa_c56920; **(C)** RT-PCR to investigate length of Assolrna spanning the region complementary to the whole *sol* operon; 1 and 2, template RNA extracted from acidogenic growth phase of *C. saccharoperbutylacetonicum* wild type with and without reverse transcriptase (RT); 3 and 4 template RNA extracted from solventogenic growth phase of *C*. *saccharoperbutylacetonicum* wild type with and without RT; 5, negative control (water); 6, positive control using genomic DNA of *C. saccharoperbutylacetonicum* wild type; **(D)** native and modified promoter of Assolrna (P_*asr*_ and P_asr_^**^ (red bases exchanged)) with −35 box, −10 box, TSS (+1) and translated end of *adc* (single letter code, *represents stop codon), predicted terminator stem loop of the *sol* operon marked with purple line (loop with second line).

Primer extension experiments with three independent primers were performed to identify the transcription start site (TSS) leading to identification of the promoter of the asRNA Assolrna (antisense to *sol* mRNA) (Figures 3A,B). The identified promoter was designated P_*asr*_ and corresponds with the sharp increase in abundance of transcripts (Figure 1). Since transcriptomic data show transcription upstream of P_*asr*_, *in silico* analysis using TraV (Dietrich et al., 2014) and different promoter prediction tools (Reese, 2001; Coppens and Lavigne, 2020) were used to identify a second TSS and the P_*asr*-is_ promoter (TTGAAA-21 nt-TCGAAT) (Figure 1). Reverse transcription PCR (RT-PCR) was performed to test for expression as continuous asRNA complementary to the complete *sol* operon during mid-exponential and stationary growth phases. It was shown that Assolrna is transcribed complementary to the *sol* operon spanning at least 3,255 bp (Figure 3C). Identification of the promoter P_*asr*_ of Assolrna encoded by *asr* showed an overlapping region with the stop codon of *adc* and a terminator (Figure 3D). An Assolrna-deficient strain was constructed by exchange of several base pairs in the promoter P_*asr*_ leading to the presumably unfunctional promoter sequence P_asr_^**^ in the promoter-exchange strain *C. saccharoperbutylacetonicum* ΔP_*asr*_ *::*P_asr_^**^ (Figure 3D).

### Growth phase dependent expression of Assolrna and *sol* mRNA

**FIGURE 4:**
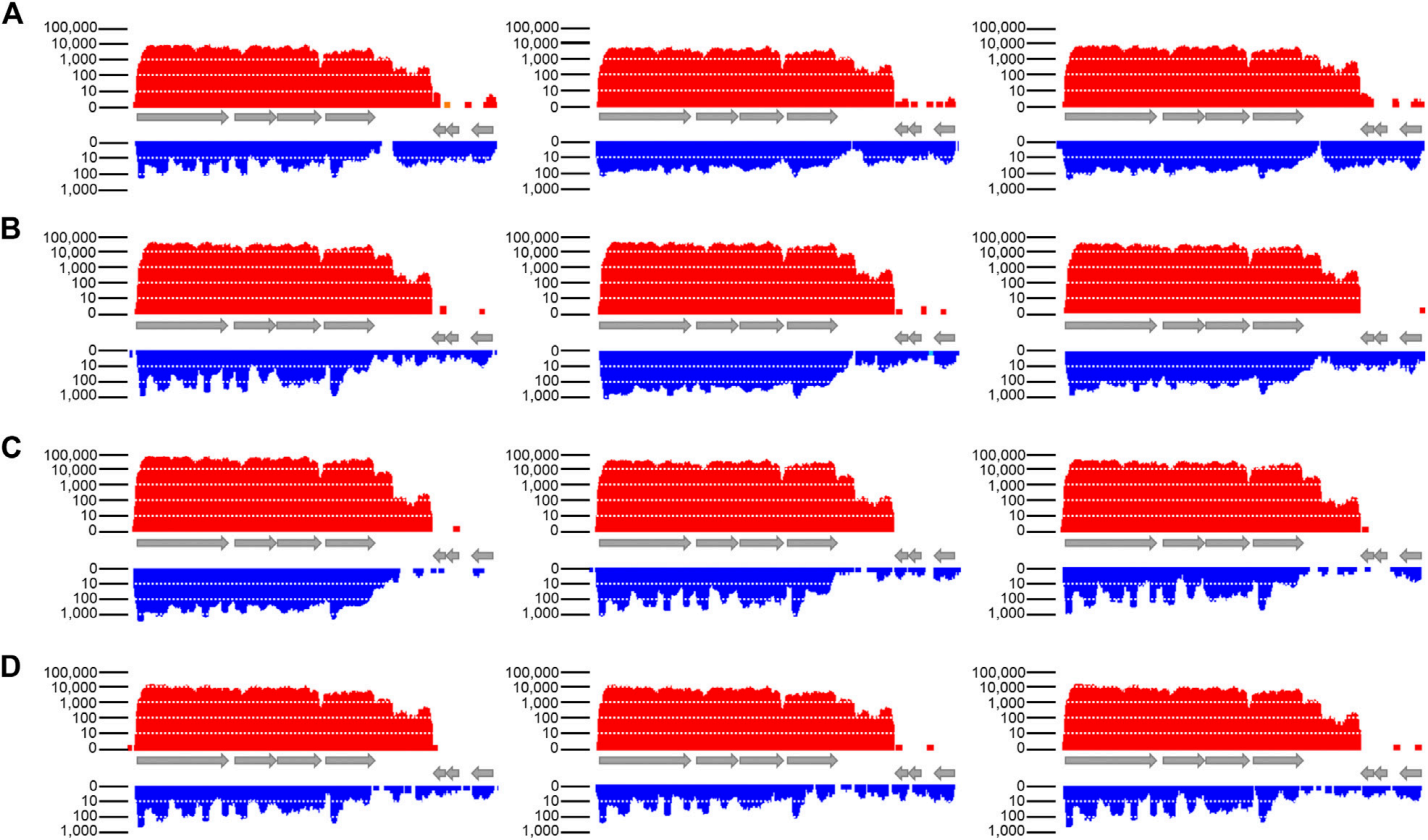
Transcriptional activity of *sol* mRNA and Assolrna as displayed in the program TraV. Columns, biological replicates; **(A)** transcription data from sampling in early exponential growth phase; **(B)** transcription data from sampling after butyrate peak; **(C)** transcription data from sampling after acetate peak; **(D)** transcription data from sampling in stationary growth phase. Red, transcriptional activity of *sol* mRNA; blue, transcriptional activity of Assolrna. Transcriptional activity is the calculated base coverage from mapped reads (Dietrich et al., 2014). Grey arrows represent genes encoding butyraldehyde dehydrogenase, CoA-transferase subunits α and β, acetoacetate decarboxylase, hypothetical protein, and two Spo0E-like sporulation regulatory proteins from left to right (Cspa_c56880–Cspa_c56940). Each column represents one biological replicate, i. e., *n* = 3. There is a sharp decrease in transcriptional activity at the end of the displayed transcript. Transcriptional activity at both ends of the figure were not cut off intentionally, they indeed ended there. This figure is modified from Baur (2022).

The transcription of *sol* mRNA and Assolrna throughout a growth experiment was assessed *via* transcriptomic analysis of biological triplicates (Baur et al., 2022). Figure 4 shows that the *sol* mRNA transcription increases for all replicates from the exponential growth phase to the acetate peak and decreases towards the stationary growth phase (Figure 4, red bars). The same is true for transcription of Assolrna (Figure 4, blue bars).

The NPKM were calculated using TraV (Dietrich et al., 2014) and are shown in Table 1 for all growth phases. The fold changes of transcription were calculated compared to transcription during the early exponential growth phase. For *sol* mRNA, they are approximately 6.4- and 9.1-fold higher at the butyrate and the acetate peaks, respectively (Table 1). During the stationary growth phase, they are reduced to 2.1-fold (Table 1). The expression profile of Assolrna is the same as for *sol* mRNA in a timely manner, whereas the fold changes range from 1.62 *via* 2.16 to 0.62 compared to Assolrna expression during the early exponential growth phase (Table 1).

**TABLE 1:** Mean NPKM values of *sol* mRNA displayed for every gene and Assolrna for different growth phases.

	Cspa_c56880	FC	Cspa_c56890	FC	Cspa_c56900	FC	Cspa_c56910	FC	Assolrna	FC
E	2,516 ± 256	1.00	2,185 ± 168	1.00	2,252 ± 102	1.00	1,581 ± 99	1.00	58 ±2	1.00
B	16,513 ± 2042	6.56	13,462 ± 1893	6.16	13,931 ±1989	6.19	10,417 ± 1,326	6.59	94 ± 8	1.62
A	22,871 ± 3,469	9.09	18,793 ± 3,308	8.60	20,071 ±4,049	8.91	15,396 ± 3,512	9.73	125 ± 57	2.16
S	6,066 ± 411	2.41	4,768 ± 304	2.18	4,716 ± 248	2.09	2,834 ± 254	1.79	36 ± 7	0.62

Samples were taking during the exponential growth phase (E), after the butyrate peak (B), after the acetate peak (A), and during the stationary growth phase (S). FC represents the fold change compared to the samples of the early exponential growth phase, *n* = 3. Cspa_c56880, bld; Cspa_c56890, ctfA; Cspa_c56900, ctfB; Cspa_c56910, adc.

### Characterization of promoter-exchange, complementation, and overexpression strains

Overexpression and complementation strains of Assolrna were constructed based on the wild type strain *C. saccharoperbutylacetonicum* N1-4(HMT) and the promoter-exchange strain *C. saccharoperbutylacetonicum* ΔP_*asr*_ *::*P_asr_^**^. The plasmid pMTL83151_Komp_P_*asr*_T harbors a truncated Assolrna under control of the native promoter P_*asr*_, whereas the plasmid pMTL83151_*asr*_P_*bgaL*_ harbors a truncated Assolrna under control of the lactose-inducible promoter P_*bgaL*_. The plasmid-based transcript was truncated compared to Assolrna encoded in the wild type chromosome because its terminator overlaps with the promoter region of the *sol* operon, i.e., P_*bld*-2_. Thereby, side effects from plasmid-based overexpression of the *sol* operon should be prevented. The plasmids as well as the vector control pMTL83151 were introduced into the wild type and the promoter-exchange strain. Growth and product formation were monitored during a growth experiment (Table 2; Supplementary Figures S1, S2). All strains based on the wild type consumed glucose completely, re-assimilated acetate and butyrate, and produced the solvents acetone, butanol, and ethanol (Supplementary Figure S1). *C. saccharoperbutylacetonicum* wild type and induced *C. saccharoperbutylacetonicum* [pMTL83151_*asr*_P_*bgaL*_] produced the highest butanol levels and the least acetone and ethanol levels (Table 2; Supplementary Figure S1). In contrast, *C. saccharoperbutylacetonicum* [pMTL83151] produced the highest acetone levels (Table 2; Supplementary Figure S1). Statistical analysis of the maximum concentrations reached by the different strains revealed that the difference in acetone and butanol formation between the wild type and *C. saccharoperbutylacetonicum* [pMTL83151], *C. saccharoperbutylacetonicum* [pMTL83151_Komp_P_*asr*_T], or non-induced *C. saccharoperbutylacetonicum* [pMTL83151_*asr*_P_*bgaL*_] was significant, but not between wild type and induced *C. saccharoperbutyl-acetonicum* [pMTL83151_*asr*_P_*bgaL*_]. Further more, the difference in butanol and acetone production between non-induced and induced *C. saccharoperbutylacetonicum* [pMTL83151_*asr*_P_*bgaL*_] is significant. The difference in acetone and butanol formation between *C. saccharoper-butylacetonicum* [pMTL83151] and *C. saccharoperbutylacetonicum* [pMTL83151_Komp_P_*asr*_T] or non-induced and induced *C. saccharo perbutylacetonicum* [pMTL83151_ *asr*_P_*bgaL*_] is significant, respectively.

**TABLE 2:** Maximal product concentrations produced with *C. saccharoperbutylacetonicum* wild type, overexpression, deletion, and complementation strains of Assolrna.

Strain	Acetate [mM]	Butyrate [mM]	Acetone [mM]	Butanol [mM]	Ethanol [mM]
*C. saccharoperbutylacetonicum* wild type	19.6 ± 2.9	9.8 ± 0.3	18.4 ± 0.4	177.5 ± 1.8	15.0 ± 0.5
*C. saccharoperbutylacetonicum* [pMTL83151]	10.7 ± 1.1	18.7 ± 0.7	28.8 ± 0.6	160.8 ± 4.6	20.0 ± 0.9
*C. saccharoperbutylacetonicum* [pMTL83151_Komp_P_*asr*_T]	13.8 ± 4.1	8.3 ± 0.8	26.0 ± 0.5	155.1 ± 8.2	23.2 ± 1.1
*C. saccharoperbutylacetonicum* [pMTL83151_*asr*_P_*bgaL*_] not induced	13.3 ± 3.6	7.6 ± 0.6	26.2 ± 1.4	154.1 ± 2.3	21.6 ± 0.7
*C. saccharoperbutylacetonicum* [pMTL83151_*asr*_P_*bgaL*_] induced	16.6 ± 1.5	7.8 ± 0.3	20.3 ± 0.3	183.6 ± 1.3	15.2 ± 0.4
*C. saccharoperbutylacetonicum* ΔP_*asr*_ *::*P_asr_^**^	14.2 ± 0.9	60.0 ± 3.0	0.5 ± 0.1	28.7 ± 2.4	4.0 ± 0.3
*C. saccharoperbutylacetonicum* ΔP_*asr*_ *::*P_asr_^**^					
[pMTL83151]	18.8 ± 1.0	55.1 ± 0.2	1.2 ± 0.1	23.3 ± 1.6	3.8 ± 0.1
*C. saccharoperbutylacetonicum* ΔP_*asr*_ *::*P_asr_^**^					
[pMTL83151_Komp_P_*asr*_T]	16.4 ± 0.7	53.5 ± 6.7	1.0 ± 0.0	29.2 ± 0.6	4.0 ± 0.0
*C. saccharoperbutylacetonicum* ΔP_*asr*_ *::*P_asr_^**^					
[pMTL83151_*asr*_P_*bgaL*_] not induced	18.3 ± 1.5	60.8 ± 1.8	0.7 ± 0.0	23.5 ± 0.9	3.9 ± 0.2
*C. saccharoperbutylacetonicum* ΔP_*asr*_ *::*P_asr_^**^					
[pMTL83151_*asr*_P_*bgaL*_] induced	21.2 ± 1.8	66.1 ± 5.2	0.2 ± 0.1	17.7 ± 1.3	3.6 ± 0.2

All products measured are rounded to one decimal place, *n* = 3.

All strains based on the promoter-exchange strain did only consume approximately half of the provided glucose, did not re-assimilate the acids, and produced very little amounts of solvents with induced *C. saccharoperbutylacetonicum* ΔP_*asr*_ *::*P_asr_^**^ [pMTL83151_*asr*_P_*bgaL*_] producing the lowest levels for all solvents (Table 2; Supplementary Figure S2). Statistical analysis was performed for the differences in butanol and acetone formation for all strains based on *C. saccharoperbutylacetonicum* ΔP_*asr*_ *::*P_asr_^**^, but no significance could be detected. In conclusion, complementation attempts resulting in phenotypical growth and solvent formation comparable to *C. saccharoperbutylacetonicum* wild type failed.

**FIGURE 5:**
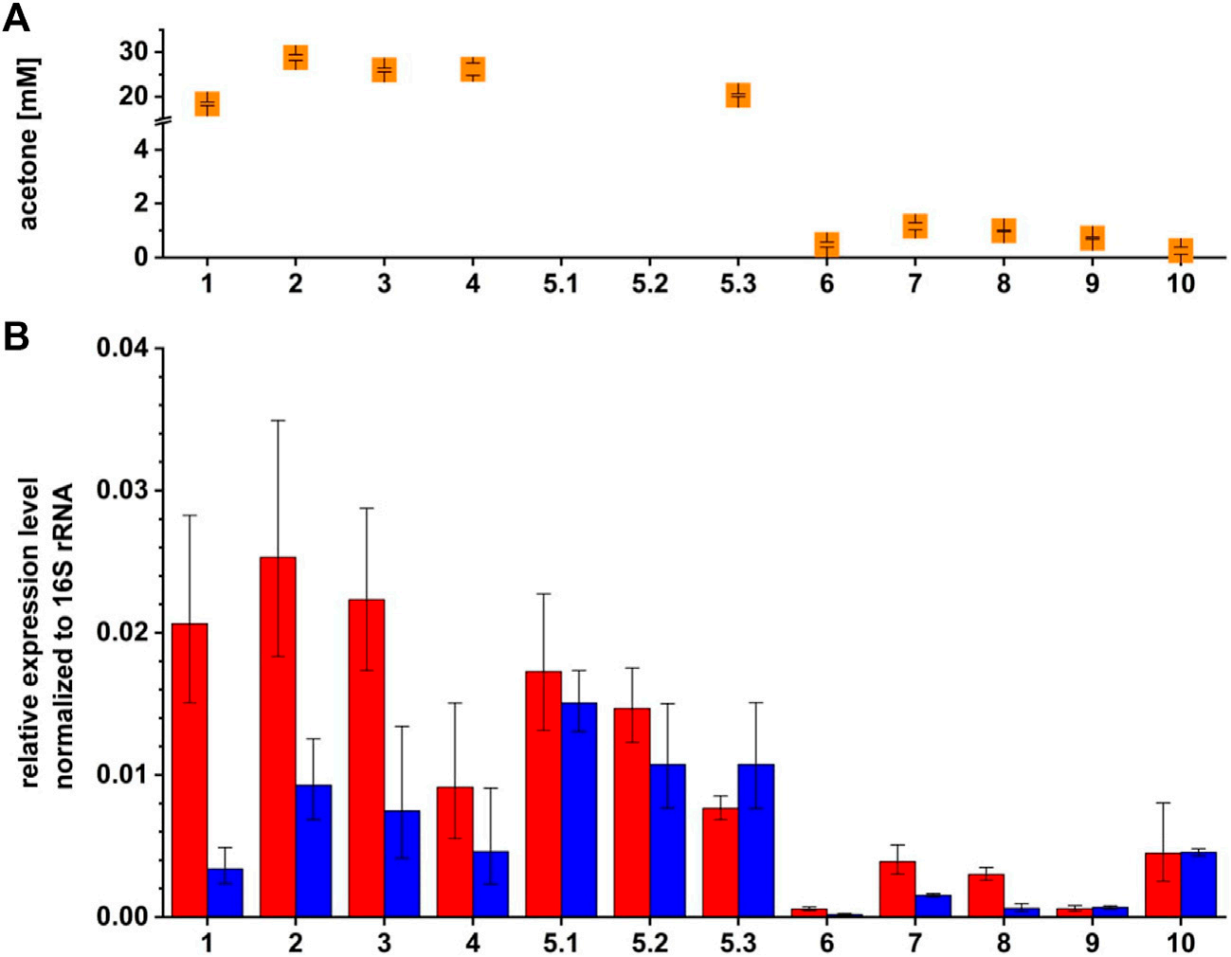
Maximal acetone concentrations and relative expression levels of *sol* mRNA and Assolrna of different *C*. *saccharoperbutylacetonicum* strains for investigation of regulation by Assolrna. **(A)** orange squares, maximal acetone concentration measured for the respective strains; **(B)** red bars, relative expression level of *sol* mRNA; blue bars, relative expression level of Assolrna normalized to expression level of 16S rRNA. RNA was extracted after 48 h of growth except for the wild type (1) because it reached the same growth phase faster than the other strains (after 34.5 h) and induced *C*. *saccharoperbutylacetonicum* [pMTL83151_*asr*_P_*bgaL*_] (5) for which RNA was extracted several times after induction (19 h of growth). This means that all samples except for 5.1 and 5.2 were prepared from the same growth phase. Numbers represent strains of which RNA was used for qRT-PCR. 1, *C. saccharoperbutylacetonicum* wild type; 2, *C. saccharoperbutylacetonicum* [pMTL83151]; 3, *C. saccharoperbutylacetonicum* [pMTL83151_Komp_P_*asr*_T]; 4, non-induced *C*. *saccharoperbutylacetonicum* [pMTL83151_*asr*_P_*bgaL*_]; 5.1, induced *C. saccharoperbutylacetonicum* [pMTL83151_*asr*_P_*bgaL*_], RNA isolated 19 h after induction; 5.2, induced *C. saccharoperbutylacetonicum* [pMTL83151_*asr*_P_*bgaL*_], RNA isolated 25.5 h after induction; 5.3, induced *C. saccharoperbutylacetonicum* [pMTL83151_*asr*_P_*bgaL*_], RNA isolated 39 h after induction; 6, *C*. *saccharoperbutylacetonicum* ΔP_*asr*_ *::*P_asr_^**^; 7, *C. saccharoperbutylacetonicum* ΔP_*asr*_ *::*P_asr_^**^ [pMTL83151]; 8, *C*. *saccharoperbutylacetonicum* ΔP_*asr*_ *::*P_asr_^**^ [pMTL83151_Komp_P_*asr*_T]; 9, non-induced *C*. *saccharoperbutylacetonicum* ΔP_*asr*_ *::*P_asr_^**^ [pMTL83151_*asr*_P_*bgaL*_]; 10, induced *C. saccharoperbutylacetonicum* ΔP_*asr*_ *::*P_asr_^**^ [pMTL83151_*asr*_P_*bgaL*_]. Error bars represent standard deviations, *n* = 3. This figure is modified from Baur (2022).

Quantitative reverse transcriptase PCR (qRT-PCR) analyses were performed to test for complementation and overexpression at transcriptional basis. Maximal acetone concentrations and transcription of *sol* mRNA and Assolrna are displayed as relative expression normalized to 16S rRNA gene expression (Figure 5). Relative expression levels of *sol* mRNA transcribed by the strains *C. saccharoperbutylacetonicum* wild type, *C. saccharoperbutylacetonicum* [pMTL83151] (vector control), and *C. saccharoperbutylacetonicum* [pMTL83151_Komp_P_*asr*_T] are comparable to each other, whereas the expression of Assolrna is lowest for the wild type and highest for the vector control strain (Figure 5B). Non-induced *C. saccharoperbutylacetonicum* [pMTL83151_*asr*_P_*bgaL*_] showed low transcription of *sol* mRNA and intermediate transcription of Assolrna. This is different for induced *C. saccharoperbutylacetonicum* [pMTL83151_*asr*_P_*bgaL*_]. Three samples taken during the growth experiment showed a decrease of *sol* mRNA transcription from 0.017 to 0.007 and stable high transcription levels of Assolrna (ranging from 0.015 to 0.011) over time (Figure 5). Transcription of *sol* mRNA was extremely low in the promoter-exchange strain *C. saccharoperbutylacetonicum* ΔP_*asr*_ *::*P_asr_^**^ for which Assolrna transcription was not detectable. Transcription of both strands was intensified when the strain harbored the vector or Assolrna under control of the native promoter, i.e., pMTL83151 and pMTL83151_ Komp_P_*asr*_T, respectively (Figure 5). Transcription of Assolrna and *sol* mRNA was equally low or high for non-induced and induced *C. saccharoperbutylacetonicum* ΔP_*asr*_ *::*P_asr_^**^ [pMTL83151_*asr*_P_*bgaL*_], respectively (Figure 5). Taking these results together, induction of Assolrna expression led to an increase in *sol* mRNA expression, but not to a change in solvent formation.

Analysis of the produced solvents together with the determined relative expression levels in the wild type background showed that acetone formation is decreased to maximal 20 mM, and butanol formation is increased to 177 mM or more for ratios of *sol* mRNA to Assolrna of 5:1 and larger or 1:1 and smaller. For ratios ranging between 3:1 and 2:1, acetone formation is increased to 26 mM or more, and butanol formation is decreased to 161 mM or less (Figure 4; Supplementary Figures S1, S2; Table 2).

### Prediction of secondary structures of Assolrna

The complex secondary structure of the native Assolrna was calculated using RNAfold web server (Gruber et al., 2008; Lorenz et al., 2011) and the result is shown in Supplementary Figure S3A. Furthermore, the secondary structures of Assolrna encoded on the plasmids pMTL83151_Komp_P_*asr*_T, pMTL83151_*asr*_P_*bgaL*_, and pMTL83151_asADC_P_*bgaL*_ were predicted and are shown in Supplementary Figures S3B–D. Predictions for the minimum free energy showed narrow structures with small loops. This differs for the predicted centroid structures with wider loops and open regions. The mountain plots are helpful for comparison of the minimum free energy with the centroid structure. The predicted native structures showed slopes, peaks, and plateaus at the same positions with differences in height (Supplemetary Figure S3A). This is different for the plasmid encoded Assolrna structures.

## Discussion

The data show that Assolrna expression increases parallel to *sol* mRNA expression during solvent production (Figure 4; Table 1) (Baur, 2022). This led to the assumption that Assolrna has a stabilizing effect on *sol* mRNA. It agrees with other stabilizing RNAs as described for *Prochlorococcus* or *Escherichia coli* (Opdyke et al., 2004; Steglich et al., 2008; Stazic et al., 2011). The stabilization occurs via different mediated effects such as mRNA stability, prevention of degradation by RNases E, J1, J2 or others, and translation initiation (Romby and Charpentier, 2010; Condon and Bechhofer, 2011; Georg and Hess, 2011; Stazic et al., 2011; Liu et al., 2015). Construction of the promoter-exchange strain *C. saccharoperbutylacetonicum* ΔP_*asr*_ *::*P_asr_^**^ resulted in a drastic decrease of solvent formation, and abolished transcription of Assolrna supporting the importance of P_*asr*_ and the very limited relevance of P_*asr*-is_ (Figures 1, 4; Supplementary Figures S1, S2; Table 2). Furthermore, the absence of Assolrna expression almost abolished *sol* mRNA transcription (Figure 4). On the one hand, the reason for this could be the mutations made to construct the non-functional P_asr_^**^ promoter were made in a region overlapping with the loop of the terminator region of the *sol* operon (Figures 1, 2). Mutations in a terminator stem loop or the polyU tail could decrease mRNA stabilization as well as successful termination (Abe and Aiba, 1996; Cisneros et al., 1996; He et al., 2020). On the other hand, most of the detected decrease in Assolrna and *sol* mRNA is probably due to the non-functional P_asr_^**^ promoter since only the loop of the terminator is modified and neither the stem nor the polyU tail. Furthermore, the induction of Assolrna expression did also increase *sol* mRNA expression. This underpins the probable stabilization mechanism for Assolrna and extends the possibility for an induction mechanism for *sol* mRNA transcription. The predicted secondary structures of *sol* mRNA revealed stem-loop structures occluding RBS and start codons of at least *bld* and *ctfA* coding regions (Supplementary Figure S4). Predicted loops complementary to the loops of *sol* mRNA were identified in secondary structure predictions of Assolrna (Supplementary Figures S3, S4).

Results of qRT-PCR show that plasmid-based Assolrna transcription is successful in terms of abundance with the inducible P_*bgaL*_ showing higher transcript levels compared to the native P_*asr*_ (Figure 5). This leads to increasing abundance in *sol* mRNA transcript in the strains based on *C. saccharoperbutylacetonicum* ΔP_*asr*_ *::*P_asr_^**^ supporting the stabilizing role of Assolrna for *sol* mRNA.

**FIGURE 6:**
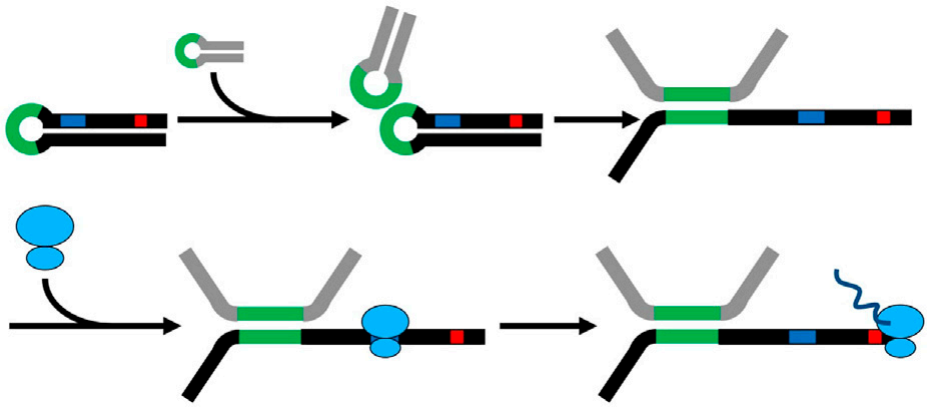
Model for regulation and enabling of translation by Assolrna. Black, *sol* mRNA with possible RBS (blue) and start codon of respective coding sequence (red); grey, Assolrna; green, possible interaction sites of *sol* mRNA and Assolrna; blue ovals, ribosome; dark blue, synthesized peptide by ribosome. The model was inspired by (Watson and Mörl, 2011) and is modified from Baur (2022).

Taking together, the results of the secondary structure predictions, solvent formation, and qRT-PCR results, a potential stabilizing mechanism is proposed (Figure 6). Loops in the Assolrna secondary structure can attach to loops of the *sol* mRNA and lead to partial melting of the *sol* mRNA structure. Then, the RBS and the start codon of the respective coding region are freed, resulting in translation and thereby, additional stabilization of the *sol* mRNA by the attached ribosomes (Figure 6). As soon as ribosomes are attached to the *sol* mRNA, Assolrna is detached from *sol* mRNA due to translational activity leading to further stabilization of the *sol* mRNA molecule. Assolrna can then alter the secondary structure of the next *sol* mRNA molecule. Another option of possible regulation by Assolrna is discussed later in combination with the unsuccessful plasmid-based complementation.

Similar mechanisms were previously described for FasX, RprA, RNAIII, and SolB (Watson and Mörl, 2011; Gupta et al., 2015; Papenfort and Vanderpool, 2015; Jones et al., 2018). The small non-coding RNA SolB which is important for regulation of solvent formation and plasmid copy number of the megaplasmid pSOL1 in *C. acetobutylicum* shows a concentration- or ratio-dependent behavior which was described by Riester (2017) and Jones et al. (2018). Binding of several ribosomes and subsequent translation of the *sol* mRNA should lead to further stabilization of the *sol* mRNA (Papenfort and Vanderpool, 2015). This is only true for physiological concentrations of Assolrna, i.e., *sol* mRNA:Assolrna ratios are higher than 2:1 (Figure 5). When the *sol* mRNA:Assolrna ratio is 1:1 or lower, Assolrna destabilizes the *sol* mRNA *via* degradation of double stranded RNA, resulting in low solvent formation. This is comparable to the proposed regulation by SolB in *C. acetobutylicum* (Jones et al., 2018). Furthermore, some reads antisense to the *sol* operon of *C. acetobutylicum*, consisting of *adhE*, *ctfA*, and *ctfB*, were identified by transcriptomics (Ralston and Papoutsakis, 2018), but the transcript and its regulatory role were not further investigated. In addition, the authors mention over 400 possible antisense interactions for other metabolic pathways (Ralston and Papoutsakis, 2018). This was also found in the transcriptomic data of *C. saccharoperbutylacetonicum*, e.g., transcripts antisense of the butyryl-CoA synthesis operon (Cspa_c04330 to Cspa_c04370) or the *pta-ack* operon (Cspa_c13010 and Cspa_c13020) (data not shown).

The vector control strains *C. saccharoperbutylacetonicum* [pMTL83151] and *C. saccharoperbutylacetonicum* ΔP_*asr*_ *::*P_asr_^**^ [pMTL83151] showed increased acetone formation and Assolrna expression, but slightly decreased butanol formation compared to the respective parental strains (Figure 4, Supplementary Figures S1, S2; Table 2). Since the only difference of these strains compared to the respective parental strains is the presence of thiamphenicol and the vector encoding the corresponding antibiotic resistance, a quorum-sensing like mechanism could be possible for regulation of Assolrna and *sol* mRNA expression with a molecule such as acetylated thiamphenicol (Feng et al., 2020). It was previously described that quorum-sensing mechanisms are involved in solvent formation and that a low-molecular weight molecule (no peptide) could restore solvent formation in a degenerated *C. saccharoperbutylacetonicum* strain (Kosaka et al., 2007).

The strains harboring truncated Assolrna under control of the native P_*asr*_ and the non-induced P_*bgaL*_ produced similar solvent levels as the respective vector controls (Table 2). Induced strains *C. saccharoperbutylacetonicum* [pMTL83151*_asr*_P_*bgaL*_] and *C. saccharoperbutylacetonicum* ΔP_*asr*_ *::*P_asr_^**^ [pMTL83151*_asr*_P_*bgaL*_] produced lowest acetone levels, thus reverting the effect resulting from the presence of the plasmid (Table 2). This backs up the destabilizing effect of high concentrations of Assolrna.

Furthermore, phenotypical complementation was not successful, and overexpression did not result in an increase of all solvents, but only of butanol (Table 2). One reason for that could be random mutagenesis somewhere else in the genome due to the use of 5-fluorocytosine as selection marker for construction of the promoter-exchange strain *C. saccharoperbutylacetonicum* ΔP_*asr*_ *::*P_asr_^**^. No compensational mechanism for the lack of Assolrna expression and, thus, lack of *sol* mRNA expression was observed as for the deletion of *bld* (Cspa_c56880) as reported in previous studies (Baur et al., 2022). Another reason for this could be regulation of *sol* mRNA expression by DNA supercoiling. In the case of the *ubiG-mccBA* operon of *C. acetobutylicum*, transcription of the antisense strand leads to coiling in the promoter region, thereby changing the angle between the −35 and the −10 region and preventing the transcription of the operon (Georg and Hess, 2011). The promoter of the *ubiG-mccBA* operon in *C. acetobutylicum* shows sequence similarity with the consensus sequence of σ^A^, with the −10 and −35 region being 18 nt apart (André et al., 2008). The *sol* operon of *C. saccharoperbutylacetonicum* has two promoters, the distal promoter P_*bld*-1_ (TTGTCT-16 nt-TAAATT-19 nt-TSS) and the proximal promoter P_*bld*-2_ (ATAACA-20 nt.TAGAAT-9 to 10 nt-TSS) (Kosaka et al., 2007). Analysis of the transcriptomic data using TraV suggests that transcription starts mainly from P_*bld*-2_ (Figure 1). Therefore, a similar mechanism could be proposed for transcription activation as described for *ubiG-mccBA* transcription prevention. Since 20 nt are quite long for the region between the −35 and −10 promoter regions, supercoiling ahead of the RNA polymerase transcribing Assolrna could alter the angle between the −35 and the −10 region of P_*bld*-2_, thus allowing the RNA polymerase transcription of the *sol* operon starting from P_*bld*-2_. This is supported by the failed phenotypical complementation and restauration of growth and solvent production to levels of the wild type using the plasmids pMTL83151_Komp_P_*asr*_T and pMTL83151_*asr*_P_*bgaL*_ (Table 2) as well as the truncated Assolrna encoded on pMTL83151_asADC_P_*bgaL*_ (data not shown). Furthermore, it can also explain, why the ratio of *sol* mRNA expression and Assolrna expression differ substantially. Higher butanol formation in the strains overproducing Assolrna in the wild type strain, i.e., *C. saccharoperbutylacetonicum* [pMTL83151_*asr*_P_*bgaL*_], could be the result from incomplete destabilization and degradation of *sol* mRNA (mainly the *bld* part) by truncated plasmid-based versions of Assolrna.

By yet unknown mechanisms, Assolrna could also recruit proteins for DNA looping mechanisms starting from an upstream enhancer site as known for σ^54^-dependent promoters eg. of RpoN regulon via NR1 in *E. coli* or of AdhA regulation *via* AdhR in *C. beijerinckii* and other clostridia (Amouyal, 2005; Nie et al., 2019; Yang et al., 2020).

In conclusion, the results obtained with the promoter-exchange strain *C. saccharoperbutylacetonicum* ΔP_*asr*_ *::*P_asr_^**^ lacking Assolrna transcription show that Assolrna expression is essential for *sol* operon expression and sufficient solvent formation to prevent acid crash. Overexpression of Assolrna in the strain *C. saccharoperbutylacetonicum* [pMTL83151_ *asr*_P_*bgaL*_] resulted in even higher butanol levels compared to *C. saccharoperbutylacetonicum* wild type. It is important to understand the mechanism of regulation by Assolrna to transfer it to other operons with antisense transcription and thereby to other native products such as acetate or butyrate. This could lead to construction of better industrial relevant strains for a range of products.

## Data Availability

The datasets presented in this study can be found in online repositories. The names of the repository/repositories and accession numbers can be found below: https://www.ncbi.nlm.nih.gov/bioproject/PRJNA774278, BioProject no. PRJNA774278 https://www.ncbi.nlm.nih.gov/bioproject/PRJNA800770, BioProject no. PRJNA800770.

## References

[B1] Abe H. Aiba H. (1996). Differential contributions of two elements of rho-independent terminator to transcription termination and mRNA stabilization. Biochimie 78, 1035–1042. 10.1016/s0300-9084(97)86727-2 9150882

[B2] Amouyal M. (2005). Gene regulation at-a-distance in *E. coli*: new insights. C. R. Biol. 328, 1–9. 10.1016/j.crvi.2004.10.002 15714875

[B3] André G. Even S. Putzer H. Burguière P. Croux C. Danchin A. (2008). S-box and T-box riboswitches and antisense RNA control a sulfur metabolic operon of *Clostridium acetobutylicum* . Nucleic Acids Res. 36, 5955–5969. 10.1093/nar/gkn601 18812398 PMC2566862

[B4] Atmadjaja A. N. Holby V. Harding A. J. Krabben P. Smith H. K. Jenkinson E. R. (2019). CRISPR-Cas, a highly effective tool for genome editing in *Clostridium saccharoperbutylacetonicum* N1-4(HMT). FEMS Microbiol. Lett. 366, fnz059. 10.1093/femsle/fnz059 30874768 PMC6491355

[B5] Bahl H. Andersch W. Gottschalk G. (1982). Continuous production of acetone and butanol by *Clostridium acetobutylicum* in a two-stage phosphate limited chemostat. Eur. J. Appl. Microbiol. Biotechnol. 15, 201–205. 10.1007/BF00499955

[B6] Baur S. T. (2022). Construction of acid-producing *Clostridium saccharoperbutylacetonicum* strains by deletion, overexpression, and interfering with genes. Ulm, Germany: University of Ulm. [dissertation]. 10.18725/OPARU-42087

[B7] Baur S. T. Markussen S. Di Bartolomeo F. Poehlein A. Baker A. Jenkinson E. R. (2022). Increased butyrate production in *Clostridium saccharoperbutylacetonicum* from lignocellulose-derived sugars. Appl. Environ. Microbiol. 88, e0241921. 10.1128/aem.02419-21 35311509 PMC9004393

[B8] Birgen C. Dürre P. Preisig H. A. Wentzel A. (2019). Butanol production from lignocellulosic biomass: revisiting fermentation performance indicators with exploratory data analysis. Biotechnol. Biofuels 12, 167. 10.1186/s13068-019-1508-6 31297155 PMC6598312

[B9] Bolger A. M. Lohse M. Usadel B. (2014). Trimmomatic: a flexible trimmer for Illumina sequence data. Bioinformatics 30, 2114–2120. 10.1093/bioinformatics/btu170 24695404 PMC4103590

[B10] Brantl S. (2007). Regulatory mechanisms employed by *cis*-encoded antisense RNAs. Curr. Opin. Microbiol. 10, 102–109. 10.1016/j.mib.2007.03.012 17387036

[B11] Cho K. H. Kim J.-H. (2015). *cis*-encoded non-coding antisense RNAs in streptococci and other low GC Gram (+) bacterial pathogens. Front. Genet. 6, 110. 10.3389/fgene.2015.00110 25859258 PMC4374534

[B12] Cisneros B. Court D. Sanchez A. Montafiez C. (1996). Point mutations in a transcription terminator, λtI, that affect both transcription termination and RNA stability. Gene 181, 127–133. 10.1016/s0378-1119(96)00492-1 8973320

[B13] Condon C. Bechhofer D. H. (2011). Regulated RNA stability in the Gram positives. Curr. Opin. Microbiol. 14, 148–154. 10.1016/j.mib.2011.01.010 21334965 PMC3078962

[B14] Coppens L. Lavigne R. (2020). Sapphire: a neural network based classifier for σ^70^ promoter prediction in *Pseudomonas* . BMC Bioinformatics 21, 415. 10.1186/s12859-020-03730-z 32962628 PMC7510298

[B15] Dietrich S. Wiegand S. Liesegang H. (2014). TraV: a genome context sensitive transcriptome browser. PLoS one 9, e93677. 10.1371/journal.pone.0093677 24709941 PMC3977867

[B16] Dürre P. (2014). Physiology and sporulation in *Clostridium* . Microbiol. Spectr. 2, TBS-0010-2012. 0010-2012. 10.1128/microbiolspec.TBS-0010-2012 26104199

[B17] Ehsaan M. Kuit W. Zhang Y. Cartman S. T. Heap J. T. Winzer K. (2016). Mutant generation by allelic exchange and genome resequencing of the biobutanol organism *Clostridium acetobutylicum* ATCC 824. Biotechnol. Biofuels 9, 4. 10.1186/s13068-015-0410-0 26732067 PMC4700727

[B18] Feng J. Zhang J. Ma Y. Feng Y. Wang S. Guo N. (2021). Renewable fatty acid ester production in *Clostridium* . Nat. Commun. 12, 4368. 10.1038/s41467-021-24038-3 34272383 PMC8285483

[B19] Feng J. Zong W. Wang P. Zhang Z.-T. Gu Y. Dougherty M. (2020). RRNPP-type quorum-sensing systems regulate solvent formation, sporulation and cell motility in *Clostridium saccharoperbutylacetonicum* . Biotechnol. Biofuels 13, 84. 10.1186/s13068-020-01723-x 32411297 PMC7206700

[B20] Fischer R. J. Helms J. Dürre P. (1993). Cloning, sequencing, and molecular analysis of the *sol* operon of *Clostridium acetobutylicum*, a chromosomal locus involved in solventogenesis. J. Bacteriol. 175, 6959–6969. 10.1128/jb.175.21.6959-6969.1993 8226639 PMC206823

[B21] Georg J. Hess W. R. (2011). *cis*-antisense RNA, another level of gene regulation in bacteria. Microbiol. Mol. Biol. Rev. 75, 286–300. 10.1128/MMBR.00032-10 21646430 PMC3122628

[B22] Green M. R. Sambrook J. (2012). Molecular cloning: A laboratory manual. Cold spring harbor. N.Y: Cold Spring Harbor Laboratory Press.

[B23] Grosse-Honebrink A. Little G. T. Bean Z. Heldt D. Cornock R. H. M. Winzer K. (2021). Development of *Clostridium saccharoperbutylacetonicum* as a whole cell biocatalyst for production of chirally pure (*R*)-1,3-butanediol. Front. Bioeng. Biotechnol. 9, 659895. 10.3389/fbioe.2021.659895 34055760 PMC8155681

[B24] Gruber A. R. Lorenz R. Bernhart S. H. Neuböck R. Hofacker I. L. (2008). The Vienna RNA websuite. Nucleic Acids Res. 36, W70–W74. 10.1093/nar/gkn188 18424795 PMC2447809

[B25] Gu Y. Feng J. Zhang Z.-T. Wang S. Guo L. Wang Y. (2019). Curing the endogenous megaplasmid in *Clostridium saccharoperbutylacetonicum* N1-4 (HMT) using CRISPR-Cas9 and preliminary investigation of the role of the plasmid for the strain metabolism. Fuel 236, 1559–1566. 10.1016/j.fuel.2018.09.030

[B26] Gupta R. K. Luong T. T. Lee C. Y. (2015). RNAIII of the *Staphylococcus aureus agr* system activates global regulator MgrA by stabilizing mRNA. Proc. Natl. Acad. Sci. U. S. A. 112, 14036–14041. 10.1073/pnas.1509251112 26504242 PMC4653210

[B27] Hanahan D. (1983). Studies on transformation of *Escherichia coli* with plasmids. J. Mol. Biol. 166, 557–580. 10.1016/S0022-2836(83)80284-8 6345791

[B28] He Z. Duan Y. Zhai W. Zhang X. Shi J. Zhang X. (2020). Evaluating terminator strength based on differentiating effects on transcription and translation. Chembiochem 21, 2067–2072. 10.1002/cbic.202000068 32180310

[B29] Hongo M. (1959). Process for producing butanol by fermentation. Washington, D.C: U.S. Patent and Trademark Office. U.S. patent No. 2,945,786A.

[B30] Huntzinger E. Boisset S. Saveanu C. Benito Y. Geissmann T. Namane A. (2005). *Staphylococcus aureus* RNAIII and the endoribonuclease III coordinately regulate *spa* gene expression. EMBO J. 24, 824–835. 10.1038/sj.emboj.7600572 15678100 PMC549626

[B31] Inoue H. Nojima H. Okayama H. (1990). High efficiency transformation of *Escherichia coli* with plasmids. Gene 96, 23–28. 10.1016/0378-1119(90)90336-P 2265755

[B32] Jones A. J. Fast A. G. Clupper M. Papoutsakis E. T. (2018). Small and low but potent: the complex regulatory role of the small RNA SolB in solventogenesis in *Clostridium acetobutylicum* . Appl. Environ. Microbiol. 84, e00597-18. 10.1128/AEM.00597-18 29728392 PMC6029083

[B33] Kosaka T. Nakayama S. Nakaya K. Yoshino S. Furukawa K. (2007). Characterization of the *sol* operon in butanol-hyperproducing *Clostridium saccharoperbutylacetonicum* strain N1-4 and its degeneration mechanism. Biosci. Biotechnol. Biochem. 71, 58–68. 10.1271/bbb.60370 17213660

[B34] Kotsanopoulos K. V. Ray R. C. Behera S. S. (2019). “Jerusalem artichoke: an emerging feedstock for bioethanol production,” in Advances in feedstock conversion technologies for alternative fuels and bioproducts: new technologies, challenges and opportunities. Editor Hosseini M. (Oxford: Woodhead Publishing), 149–161.

[B35] Langmead B. Salzberg S. L. (2012). Fast gapped-read alignment with Bowtie 2. Nat. Methods 9, 357–359. 10.1038/nmeth.1923 22388286 PMC3322381

[B36] Liu N. Niu G. Xie Z. Chen Z. Itzek A. Kreth J. (2015). The *Streptococcus mutans irvA* gene encodes a trans-acting riboregulatory mRNA. Mol. Cell 57, 179–190. 10.1016/j.molcel.2014.11.003 25574948 PMC5663275

[B37] Lorenz R. Bernhart S. H. Höner Zu Siederdissen C. Tafer H. Flamm C. Stadler P. F. (2011). ViennaRNA package 2.0. Algorithms Mol. Biol. 6, 26. 10.1186/1748-7188-6-26 22115189 PMC3319429

[B38] Love M. I. Huber W. Anders S. (2014). Moderated estimation of fold change and dispersion for RNA-seq data with DESeq2. Genome Biol. 15, 550. 10.1186/s13059-014-0550-8 25516281 PMC4302049

[B39] Montoya Solano J. D. (2013). Metabolic engineering of the Colombian strain Clostridium sp. IBUN 158B in order to improve the bioconversion of glycerol into 1,3-propanediol. Ulm, Germany: University of Ulm. [dissertation]. 10.18725/OPARU-2603

[B40] Morris K. V. Mattick J. S. (2014). The rise of regulatory RNA. Nat. Rev. Genet. 15, 423–437. 10.1038/nrg3722 24776770 PMC4314111

[B41] Mortazavi A. Williams B. A. McCue K. Schaeffer L. Wold B. (2008). Mapping and quantifying mammalian transcriptomes by RNA-Seq. Nat. Methods 5, 621–628. 10.1038/nmeth.1226 18516045 PMC13303166

[B42] Nie X. Dong W. Yang C. (2019). Genomic reconstruction of σ^54^ regulons in *Clostridiales* . BMC Genomics 20, 565. 10.1186/s12864-019-5918-4 31288763 PMC6615313

[B43] Ogata S. Hongo M. (1979). Bacteriophages of the genus *Clostridium* . Adv. Appl. Microbiol. 25, 241–273. 10.1016/s0065-2164(08)70152-7 397738

[B44] Opdyke J. A. Fozo E. M. Hemm M. R. Storz G. (2011). RNase III participates in GadY-dependent cleavage of the *gadX-gadW* mRNA. J. Mol. Biol. 406, 29–43. 10.1016/j.jmb.2010.12.009 21147125 PMC3030618

[B45] Opdyke J. A. Kang J.-G. Storz G. (2004). GadY, a small-RNA regulator of acid response genes in *Escherichia coli* . J. Bacteriol. 186, 6698–6705. 10.1128/JB.186.20.6698-6705.2004 15466020 PMC522195

[B46] Papenfort K. Vanderpool C. K. (2015). Target activation by regulatory RNAs in bacteria. FEMS Microbiol. Rev. 39, 362–378. 10.1093/femsre/fuv016 25934124 PMC4542691

[B47] Patro R. Duggal G. Love M. I. Irizarry R. A. Kingsford C. (2017). Salmon provides fast and bias-aware quantification of transcript expression. Nat. Methods 14, 417–419. 10.1038/nmeth.4197 28263959 PMC5600148

[B48] Poehlein A. Krabben P. Dürre P. Daniel R. (2014). Complete genome sequence of the solvent producer *Clostridium saccharoperbutylacetonicum* strain DSM 14923. Genome Announc. 2, e01056-14. 10.1128/genomeA.01056-14 25323722 PMC4200160

[B49] Poehlein A. Montoya Solano J. D. Flitsch S. K. Krabben P. Winzer K. Reid S. J. (2017). Microbial solvent formation revisited by comparative genome analysis. Biotechnol. Biofuels 10, 58. 10.1186/s13068-017-0742-z 28286553 PMC5343299

[B50] R Core Team (2020). R: A language and environment for statistical computing. Vienna, Austria: R Foundation for Statistical Computing.

[B51] Ralston M. T. Papoutsakis E. T. (2018). RNAseq-based transcriptome assembly of *Clostridium acetobutylicum* for functional genome annotation and discovery. AIChE J. 64, 4271–4280. 10.1002/aic.16396

[B52] Reese M. G. (2001). Application of a time-delay neural network to promoter annotation in the *Drosophila melanogaster* genome. Comput. Chem. 26, 51–56. 10.1016/S0097-8485(01)00099-7 11765852

[B53] Riester E. (2017). Tracking the elusive function of the regulatory RNA SolB in solventogenesis and its relation to the RNA-binding protein Hfq in *Clostridium acetobutylicum* . Ulm, Germany: University of Ulm. [dissertation]. 10.18725/OPARU-4439

[B54] Romby P. Charpentier E. (2010). An overview of RNAs with regulatory functions in Gram-positive bacteria. Cell. Mol. Life Sci. 67, 217–237. 10.1007/s00018-009-0162-8 19859665 PMC11115938

[B55] Schmittgen T. D. Livak K. J. (2008). Analyzing real-time PCR data by the comparative C_T_ method. Nat. Protoc. 3, 1101–1108. 10.1038/nprot.2008.73 18546601

[B56] Soneson C. Love M. I. Robinson M. D. (2015). Differential analyses for RNA-seq: transcript-level estimates improve gene-level inferences. F1000Res. 4, 1521. 10.12688/f1000research.7563.1 26925227 PMC4712774

[B57] Stazic D. Lindell D. Steglich C. (2011). Antisense RNA protects mRNA from RNase E degradation by RNA-RNA duplex formation during phage infection. Nucleic Acids Res. 39, 4890–4899. 10.1093/nar/gkr037 21325266 PMC3113571

[B58] Steglich C. Futschik M. E. Lindell D. Voss B. Chisholm S. W. Hess W. R. (2008). The challenge of regulation in a minimal photoautotroph: non-coding RNAs in *Prochlorococcus* . PLoS Genet. 4, e1000173. 10.1371/journal.pgen.1000173 18769676 PMC2518516

[B59] Thomason M. K. Storz G. (2010). Bacterial antisense RNAs: how many are there, and what are they doing? Annu. Rev. Genet. 44, 167–188. 10.1146/annurev-genet-102209-163523 20707673 PMC3030471

[B60] Wang M.-Y. Tsai Y.-L. Olson B. H. Chang J.-S. (2008). Monitoring dark hydrogen fermentation performance of indigenous *Clostridium butyricum* by hydrogenase gene expression using RT-PCR and qPCR. Int. J. Hydrogen Energy 33, 4730–4738. 10.1016/j.ijhydene.2008.06.048

[B61] Watson J. D. Mörl M. (2011). Molekularbiologie. Hallbergmoos, Germany: Pearson Studium.

[B62] Wirth S. Dürre P. (2021). Investigation of putative genes for the production of medium-chained acids and alcohols in autotrophic acetogenic bacteria. Metab. Eng. 66, 296–307. 10.1016/j.ymben.2021.04.010 33894339

[B63] Yang B. Nie X. Gu Y. Jiang W. Yang C. (2020). Control of solvent production by sigma-54 factor and the transcriptional activator AdhR in *Clostridium beijerinckii* . Microb. Biotechnol. 13, 328–338. 10.1111/1751-7915.13505 31691520 PMC7017808

[B64] Zhu A. Ibrahim J. G. Love M. I. (2019). Heavy-tailed prior distributions for sequence count data: removing the noise and preserving large differences. Bioinformatics 35, 2084–2092. 10.1093/bioinformatics/bty895 30395178 PMC6581436

