# Supplementary material for: Modulation of sol mRNA expression by the long non-coding RNA Assolrna in Clostridium saccharoperbutylacetonicum affects solvent formation
Source: Front Genet. 2022 Aug 11;13:966643. doi: 10.3389/fgene.2022.966643 (PMC9402939; doi:10.3389/fgene.2022.966643)
Supplement: Supplementary file 1 [file DataSheet1.docx]

Supplementary Material


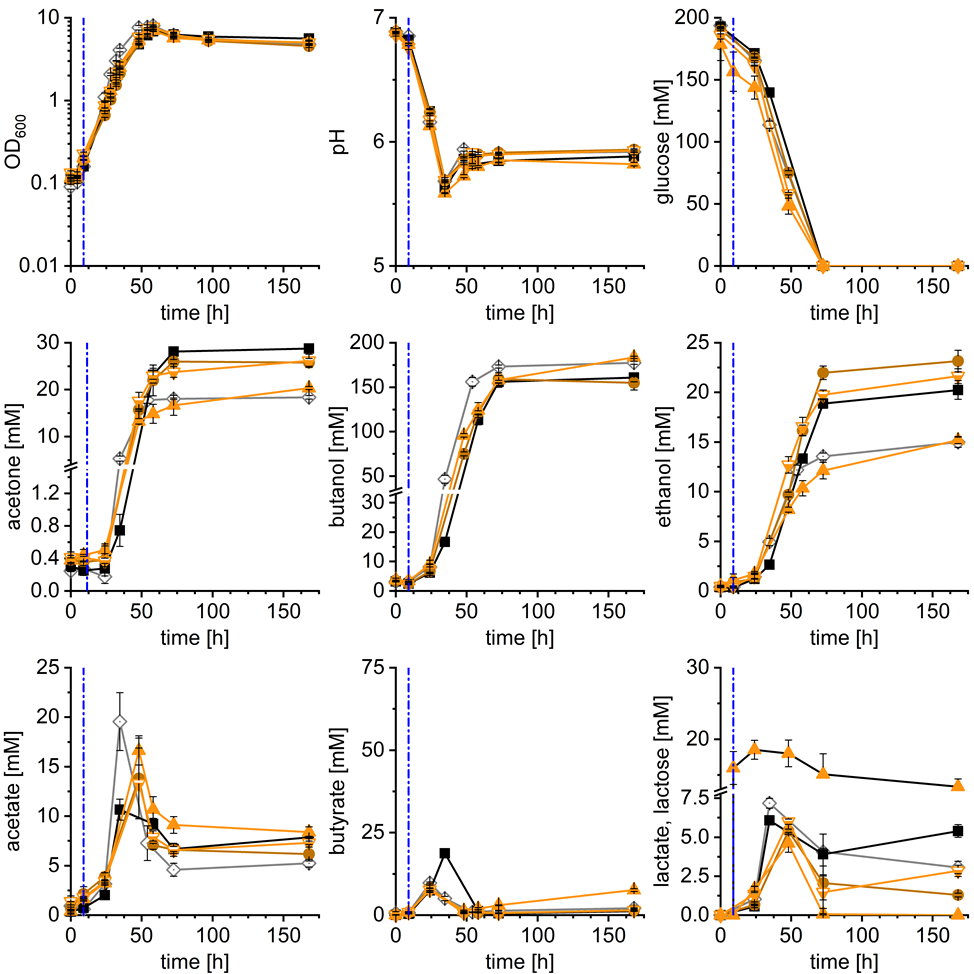


**Figure S1. Growth experiment using strains based on *C. saccharoperbutylacetonicum* for investigation of regulation by Assolrna.** OD_600_, pH, and concentrations of substrates and products are given over the course of the experiment. Grey diamond with dot, *C. saccharoperbutylacetonicum* wild type; black squares, *C. saccharoperbutylacetonicum* [pMTL83151]; brown circles, *C. saccharoperbutylacetonicum* [pMTL83151_Komp_P*_asr_*T]; orange triangles down half filled, non-induced *C. saccharoperbutylacetonicum* [pMTL83151_*asr*_P*_bgaL_*]; orange triangles up, induced *C. saccharoperbutylacetonicum* [pMTL83151_*asr*_P*_bgaL_*]; black line with orange triangles, lactose concentration; dash-dot line, induction with 20 mM lactose. Error bars represent standard deviations, n=3. This figure is modified from Baur (2022).

**
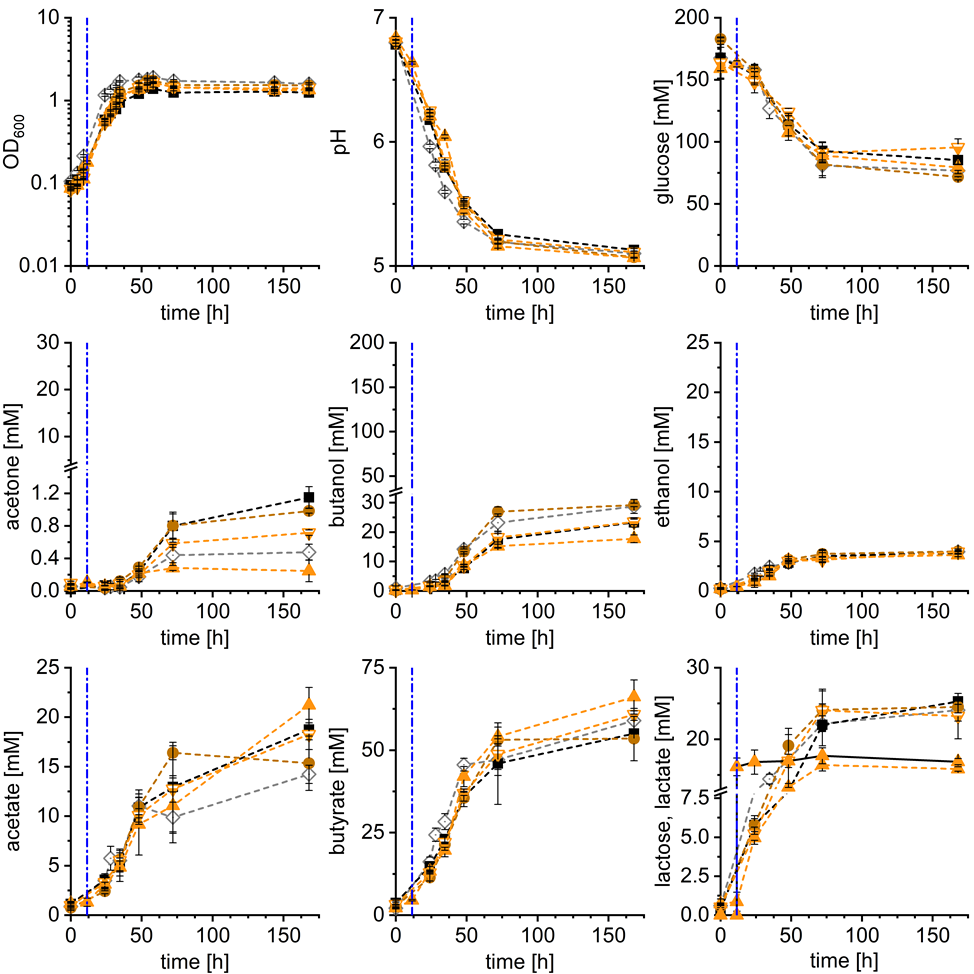
**

**Figure S2. Growth experiment using strains based on *C. saccharoperbutylacetonicum* ΔP*_asr_::*P*_asr_*^**^ for investigation of regulation by Assolrna.** OD_600_, pH, and concentrations of substrates and products are given over the course of the experiment. Grey diamonds with dot, *C. saccharoperbutylacetonicum* ΔP*_asr_::*P*_asr_*^**^; black squares, *C. saccharoperbutylacetonicum* ΔP*_asr_::*P*_asr_*^**^ [pMTL83151]; brown circles, *C. saccharoperbutylacetonicum* ΔP*_asr_::*P*_asr_*^**^ [pMTL83151_Komp_P*_asr_*T]; orange triangles down half filled, non-induced *C. saccharoperbutylacetonicum* ΔP*_asr_::*P*_asr_*^**^ [pMTL83151_*asr*_P*_bgaL_*]; orange triangles up, induced *C. saccharoperbutylacetonicum* ΔP*_asr_::*P*_asr_*^**^ [pMTL83151_*asr*_P*_bgaL_*]; black line with orange triangles, lactose concentration; dash-dot line, induction with 20 mM lactose. Error bars represent standard deviations, n=3. This figure is modified from Baur (2022).


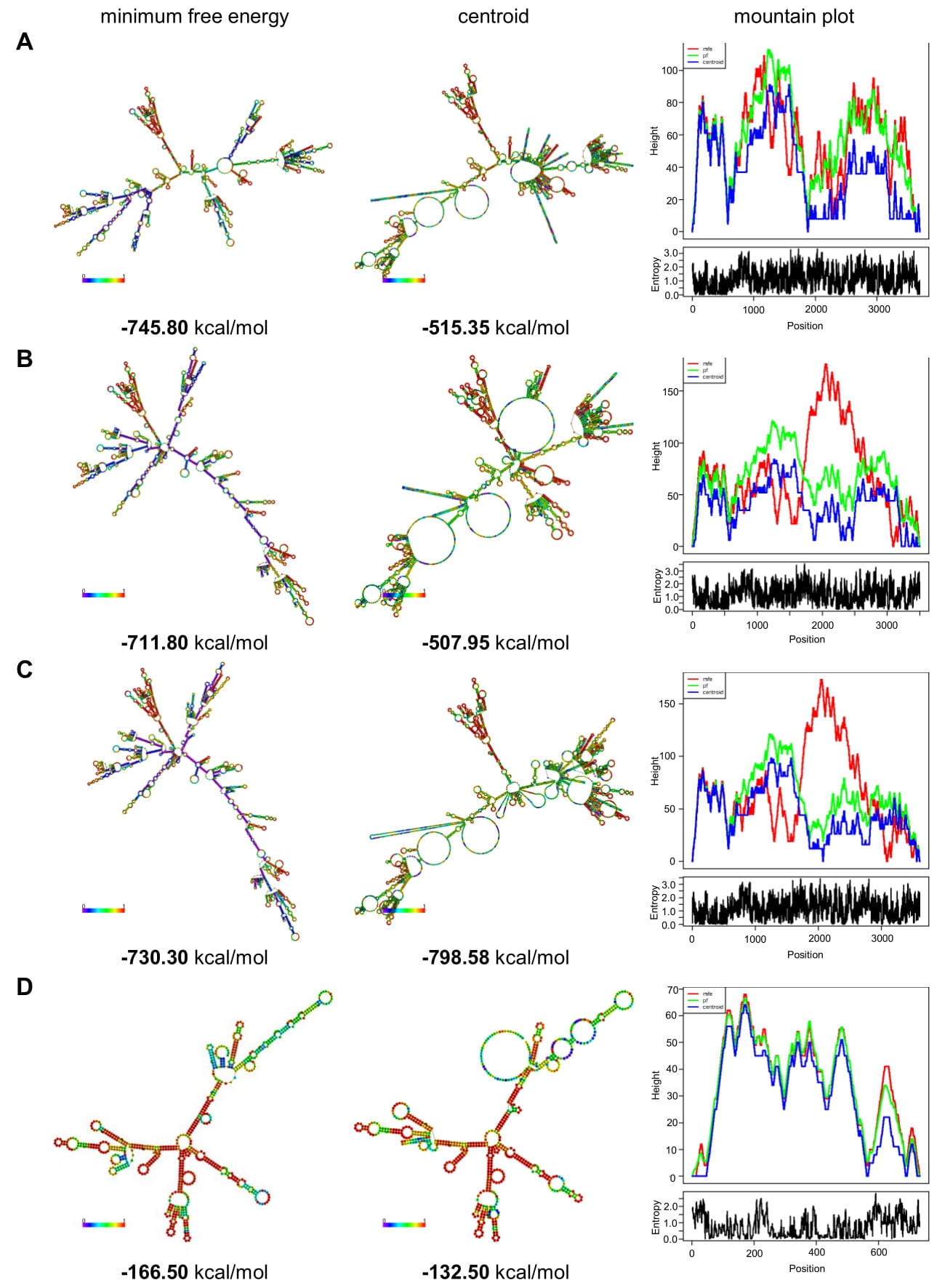


**Figure S3. Predicted secondary structures of Assolrna.** Purple colored bases, base pair probability equals 0; red colored bases, base pair probability equals 1. Mountain plots show secondary structure as height versus position: red line, minimum free energy structure; blue line, centroid structure; green line, partition function; black line, positional entropy versus position. **A**, secondary structures and mountain plot for native Assolrna; **B**, secondary structures and mountain plot for Assolrna subcloned in pMTL83151_Komp_P*_asr_*T; **C**, secondary structures and mountain plot for Assolrna subcloned in pMTL83151_*asr*_P*_bgaL_*; **D**, secondary structures and mountain plot for Assolrna subcloned in pMTL83151_asADC_P*_bgaL_*. This figure is modified from Baur (2022).


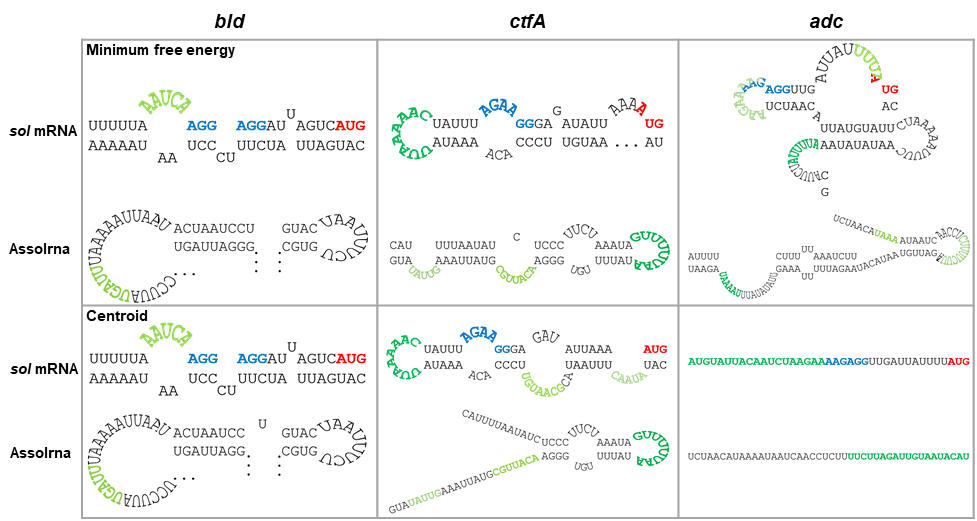


**Figure S4. Possible interactions of Assolrna and *sol* mRNA based on predicted secondary structures. Blue**, possible RBS; **red**, start codon of respective coding sequence; **green**, possible interaction sites of *sol* mRNA and Assolrna. Segments showing predicted secondary structures of *sol* mRNA and Assolrna upstream of ***bld***, gene encoding butyraldehyde dehydrogenase (Cspa_c56880), ***ctfA***, CoA-transferase subunit α (Cspa_c56890); ***adc***, acetoacetate decarboxylase (Cspa_c56910) as minimum free energy and centroid prediction of secondary structure (Figure 5). This figure is modified from Baur (2022).

**Table S1.** Bacterial strains and plasmids used in this study.

| **Strain or plasmid** | **Characteristics** | **Origin** |
| --- | --- | --- |
| *E. coli* XL1-Blue MRF’ | Δ(*mcrA*)183 Δ(*mrr-hsdRMS-mcrBC*)173 *endA1 supE44 thi-1 recA1 gyrA6 relA1 lac* [F^-^ *proAB lacI^q^Z*ΔM15 Tn*10* (Tet*^r^*) | Agilent Technologies Inc. (Santa Clara, CA, USA) |
| *E. coli* DH5*α* | F^-^ *ϕ80 lacZ*ΔM15 Δ(*lacZYA*-*argF*) U169 *recA1 endA1 hsdR17* (r^-^*_k_* , m^-^*_k_*) *phoA supE44 thi-1 gyrA96 relA1 λ^-^* | Thermo Fisher Scientific Inc. (Waltham, MA, USA) |
| *E. coli* CA434 | hsdS20 (r^-^_B_, m^-^_B_) *supE44 thi-1 recAB ara-14 leuB5proA2 lacY1 galK rpsL20 (Str^R^) xyl-5 mtl-1* with conjugative plasmid R702 (Sm^R^, Su^R^, Hg^R^, Tra^+^, Mob^+^) | Des Purdy et al., 2002 |
| *C. saccharoperbutylacetonicum* N1-4(HMT) (DSM 14923) | type strain | DSMZ*^a^* (Brunswick, Germany) |
| *C. saccharoperbutylacetonicum* ΔP*_asr_::*P*_asr_*^**^ | *C. saccharoperbutylacetonicum* N1-4(HMT), promoter region P*_asr_* exchanged with unfunctional sequence P*_asr_*^**^ | Baur, 2022 Klicken oder tippen Sie hier, um Text einzugeben. |
| pDrive | Amp^R^, Kan^R^, pUC *ori*^-^, phage f1 *ori*^-^, *lacZα* | Qiagen N.V., Hilden, Germany |
| pDrive_11 | pDrive, fragment amplified using primers Fwd_1_solregpotRNA and Rev_1_solregpotRNA | This study |
| pDrive_12 | pDrive, fragment amplified using primers Fwd_1_solregpotRNA and Rev_2_solregpotRNA | This study |
| pDrive_13 | pDrive, fragment amplified using primers Fwd_1_solregpotRNA and Rev_3_solregpotRNA | This study |
| pDrive_21 | pDrive, fragment amplified using primers Fwd_2_solregpotRNA and Rev_1_solregpotRNA | This study |
| pDrive_22 | pDrive, fragment amplified using primers Fwd_2_solregpotRNA and Rev_2_solregpotRNA | This study |
| pDrive_recA | pDrive, fragment amplified using LHA_fwd_PromRNA and RHA_rev_PromRNA (knock-out cassette for P*_asr_* replaced by P*_asr_*^*^) | This study |
| pMTL83151 | *catP*, ColE1 *ori*^-^, *lacZα*, pCB102 *ori^+^*, *traJ* | Heap et al., 2009 |
| pMTL83251 | *ermB*, ColE1 *ori*^-^, *lacZα*, pCB102 *ori^+^*, *traJ* | Heap et al., 2009 |
| pMTL-SC7515 | *catP*, ColE1 *ori*^-^, pIM13 *ori^+^*, *traJ*, *codA* (gene encoding cytosine deaminase) under control of P*_fac_* | Ehsaan et al., 2016 |
| pMTLSC7515-Em | pMTL-SC7515, *catP* exchanged using *ermB* from pMTL83251 | This study |
| pMTL-PromoterRNA/ pMTLSC7515-Em-recA | pMTLSC7515-Em, knock-out cassette for P*_asr_* replaced by P*_asr_*^*^ Sequence of P*_asr_*^*^ (red indicates mutated bases): CCATAGTGGTTATTCTATATTATCCCTA CTTCAAGG Sequence of P*_asr_*** (identified mutation in promoter-exchange strain, red indicated mutated bases): TCGTAGTGGTTATTCTATATTATCCCTA CTTCAAGG | This study |
| pMTL83151_gusA_P*_bgaL_* | pMTL83151, *gusA* from *E. coli* under control of P*_bgaL_* with activator *bgaR* originating from *Clostridium perfringens* | Beck et al., 2020 |
| pMTL83151_gusA_P*_fac_* | pMTL83151, *gusA* from *E. coli* under control of P*_fac_* originating from *Clostridium pasteurianum* with *lac* operator and repressor *lacI* originating from *E. coli* | Beck et al., 2020 |
| pMTL83151_gusA_P*_tet_* | pMTL83151, *gusA* from *E. coli* under control of P*_tet_* with repressor *tetR* originating from *Bacillus subtilis* | Beck et al., 2020 |
| pMTL83151_Komp_P*_asr_* | pMTL83151, fragment amplified using primers pMTL8-151_Komp_Pasr_fwd and pMTL8-151_Komp_Pasr_rev (truncated Assolrna) | Baur, 2022 |
| pMTL83151_Komp_P*_asr_*T | pMTL83151_Komp_P*_asr_*, fragment amplified using primers Tfdx_fwd and Tfdx_rev (terminator T*_fdx_*) | Baur, 2022 |
| pMTL83151_*asr*_P*_tet_* | pMTL83151_gusA_P*_tet_*, *gusA* exchanged by fragment amplified using primers asr_XhoI_fwd and asr_NheI_rev (truncated Assolrna) | Baur, 2022 |
| pMTL83151_*asr*_P*_bgaL_* | pMTL83151_*asr*_P*_tet_*, P*_tet_* exchanged by P*_bgaL_* | Baur, 2022 |
| pMTL83151_asADC_P*_asr_*T | pMTL83151_Komp_P*_asr_*T, Assolrna exchanged by fragment amplified using primers asADC_Pasr_fwd and asADC_Pasr_rev (truncated Assolrna) | This studyKlicken oder tippen Sie hier, um Text einzugeben. |

**Table S2. Primers used in this study.**

| **Name** | **Sequence** | **Purpose** |
| --- | --- | --- |
| Fwd_1_solregpotRNA | GAAATGTGCTTAAGTTAAGTGAACATCG | PEX to find the transcription start site of Assolrna |
| Fwd_2_solregpotRNA | GTTAAATATATAGGTGGTATCCCGCTGG |  |
| Rev_1_solregpotRNA | CCTGTATTAGAGATTGTATCAGCATCTCATATCC |  |
| Rev_2_solregpotRNA | GGTGCTTGGACTGGAAGTGCACGTCTG |  |
| Rev_3_solregpotRNA | GCCATGCACTAGCTCCTCTTGCTG |  |
| ermC-FseI_fwd | ACAGGCCGGCCATGCACTAATG | Colony PCR for identification of constructed pMTLSC7515-Em |
| ermC-nachPmeI_rev | TCCGGCAAACAAACCACCGC |  |
| M13F | GTAAAACGACGGCCAG | Colony PCR for identification of constructed plasmids for PEX |
| M13R | AACAGCTATGACCATG |  |
| LHA_fwd_PromRNA | ttttttGTTTAAACGCATATGATGTATCTAGATAATGAACCCG | PCR for construction of knock-out cassette for P*_asr_* replaced by P*_asr_*^*^ in  pMTL-PromoterRNA |
| LHA_rev_PromRNA | CTACTTCCATAGTGGTTATTCTATATTATCCCTACTTCAAGGAAAGATAATC* |  |
| RHA_fwd_PromRNA | GATTATCTTTCCTTGAAGTAGGGATAATATAGAATAACCACTATGGAAG* |  |
| RHA_rev_PromRNA | ttttttGTTTAAACCAAATATGAGGATAAATTTAGATC |  |
| fD1 | AGAGTTTGATGCTCAG | PCR and sequencing of 16S rDNA sequence |
| rP2 | ACGGCTACCTCGACTT |  |
| pMTL-PR-codA_fwd | TTATCCGCTCACAATTCC | PCR to test for presence of pMTL-PromoterRNA |
| pMTL-PR-codA_rev | CCTTTAAACTAAGCTCGC |  |
| pMTL-PR_fwd | TAAATATGAGCGAAGCGGTT | Screening for genomic integration and excision of pMTL-PromoterRNA |
| pMTL-PR_rev | GGGATTTTGGTCATGAGATT |  |
| assolrna-genom_fwd | GGTAAGCCAAGAATTTGTGA | Screening for genomic integration of  pMTL-PromoterRNA, PCR and sequencing of P*_asr_*/P*_asr_*^**^ region |
| assolrna-genom_rev | AATATATAGGATCCCG |  |
| pMTL8-51_Komp_Pasr_fwd | CTCGGTACCCGGGGATCCTCTAGAGGTCGACGCTATAAGCAATGCCGTAC | Cloning of pMTL83151_Komp_P*_asr_* |
| pMTL8-51_Komp_Pasr_rev | TGCCAAGCTTGCATGTCTGCAGGCCCTCGAGGAGTAAAAAGGGGACACAAAG |  |
| Tfdx_fwd | CGAATTCGAGCTCGGTACCCATTTTCTTTTCTGTAAATTTCTTTCTATTC | Cloning of pMTL83151_Komp_P*_asr_*T |
| Tfdx_rev | TCGACCTCTAGAGGATCCCCATAAAAATAAGAAGCCTGCATTTG |  |
| asr_XhoI_fwd | ACAACACTCGAGATCATGTACAACCTTAGG | Cloning of pMTL83151_*asr*_P*_tet_* |
| asr_NheI_rev | ACAACAGCTAGCACACGCTAGTTTCTATAAC |  |
| asADC_Pasr_fwd | TTATGGGGATCCTCTAGAGGTCGACATGTTAGAAAGTGAAGTATCTAAAC | Cloning of pMTL83151_asADC_ P*_asr_*T |
| asADC_Pasr_rev | GCTTGCATGTCTGCAGGCCCTCGAGTAAAAAGGGGACACAAAG |  |
| RT_asr_fwd | GCTTGGTCAGGAGATAAC | RT-PCR to confirm length of Assolrna |
| RT_ig670_rev | CCTTAGGTGTTCCAAGAG |  |
| 16SF_qPCR | AGCGTTGTCCGGATTTACTG | Primer efficiency test and qRT-PCR targeting 16S rRNA |
| 16SR_qPCR | TTCGCCACTGGTATTCTTCC |  |
| AdhEF_qPCR | AGGAGATAACGGGCTTACAG | Primer efficiency test and qRT-PCR targeting *bld* |
| AdhER_qPCR | TTCGACAGCAAAAGCAACAC |  |
| AdcF_qPCR | CAACAATGGGATACAAGCACG | Primer efficiency test and qRT-PCR targeting *adc* |
| AdcR_qPCR | CACTTCCAGTCCAAGCACC |  |
| pMTL83151_PoI_fwd | CGCTGTATCCATATGACCA | Sequencing of pMTL83151_Komp_P*_asr_*, pMTL83151_Komp_P*_asr_*T, pMTL83151_*asr*_P*_tet_*, and pMTL83151_*asr*_P*_bgaL_* |
| pMTL83151_seq_gusA_ PbgaL | CACAATTAGCAACACAGG |  |
| seq_asr1_fwd | CAGCAAGTGGAGTTGTAA |  |
| seq_asr2_fwd | AATCTACTAATACGCCAGG |  |
| seq_asr3_fwd | AGTTGTAAAGCCTTCTGC |  |
| seq_asr4_fwd | TTTTTTAGCGCCTGGATG |  |
| Adc_vitro_Tem1_fwd | TCCCACTTACTGCTAAAG |  |

*: This primer sequences should have let to the P*_asr_*^**^ -35 region “CCATAG”. No clone with this sequence was obtained. The only identified, mutated clone had the P*_asr_*^**^ promoter sequence “TCGTAG” (Figure 2).

**References**

Baur, S. T. (2022) Construction of acid-producing *Clostridium saccharoperbutylacetonicum* strains by deletion, overexpression, and interfering with genes. [dissertation]. [Ulm, Germany]: University of Ulm. doi: 10.18725/OPARU-42087

Beck, M. H., Flaiz, M., Bengelsdorf, F. R., and Dürre, P. (2020). Induced heterologous expression of the arginine deiminase pathway promotes growth advantages in the strict anaerobe *Acetobacterium woodii*. Appl Microbiol Biotechnol 104, 687–699. doi: 10.1007/s00253-019-10248-9

Des Purdy, O'Keeffe, T. A. T., Elmore, M., Herbert, M., McLeod, A., Bokori-Brown, M., et al. (2002). Conjugative transfer of clostridial shuttle vectors from *Escherichia coli* to *Clostridium difficile* through circumvention of the restriction barrier. Mol Microbiol 46, 439–452. doi: 10.1046/j.1365-2958.2002.03134.x

Ehsaan, M., Kuit, W., Zhang, Y., Cartman, S. T., Heap, J. T., Winzer, K., et al. (2016). Mutant generation by allelic exchange and genome resequencing of the biobutanol organism *Clostridium acetobutylicum* ATCC 824. Biotechnol Biofuels 9, 4. doi: 10.1186/s13068-015-0410-0

Heap, J. T., Pennington, O. J., Cartman, S. T., and Minton, N. P. (2009). A modular system for *Clostridium* shuttle plasmids. J Microbiol Methods 78, 79–85. doi: 10.1016/j.mimet.2009.05.004
